# A review on lignin pyrolysis: pyrolytic behavior, mechanism, and relevant upgrading for improving process efficiency

**DOI:** 10.1186/s13068-022-02203-0

**Published:** 2022-10-11

**Authors:** Xinyu Lu, Xiaoli Gu

**Affiliations:** Jiangsu Co-Innovation Center of Efficient Processing and Utilization of Forest Resources, College of Chemical Engineering, Nanjing Forestry University, Nanjing, 210037 China

**Keywords:** Lignin, Pyrolysis, Phenolic compounds, Aromatic hydrocarbons

## Abstract

Lignin is a promising alternative to traditional fossil resources for producing biofuels due to its aromaticity and renewability. Pyrolysis is an efficient technology to convert lignin to valuable chemicals, which is beneficial for improving lignin valorization. In this review, pyrolytic behaviors of various lignin were included, as well as the pyrolytic mechanism consisting of initial, primary, and charring stages were also introduced. Several parallel reactions, such as demethoxylation, demethylation, decarboxylation, and decarbonylation of lignin side chains to form light gases, major lignin structure decomposition to generate phenolic compounds, and polymerization of active lignin intermediates to yield char, can be observed through the whole pyrolysis process. Several parameters, such as pyrolytic temperature, time, lignin type, and functional groups (hydroxyl, methoxy), were also investigated to figure out their effects on lignin pyrolysis. On the other hand, zeolite-driven lignin catalytic pyrolysis and lignin co-pyrolysis with other hydrogen-rich co-feedings were also introduced for improving process efficiency to produce more aromatic hydrocarbons (AHs). During the pyrolysis process, phenolic compounds and/or AHs can be produced, showing promising applications in biochemical intermediates and biofuel additives. Finally, some challenges and future perspectives for lignin pyrolysis have been discussed.

## Introduction

**Fig. 1:**
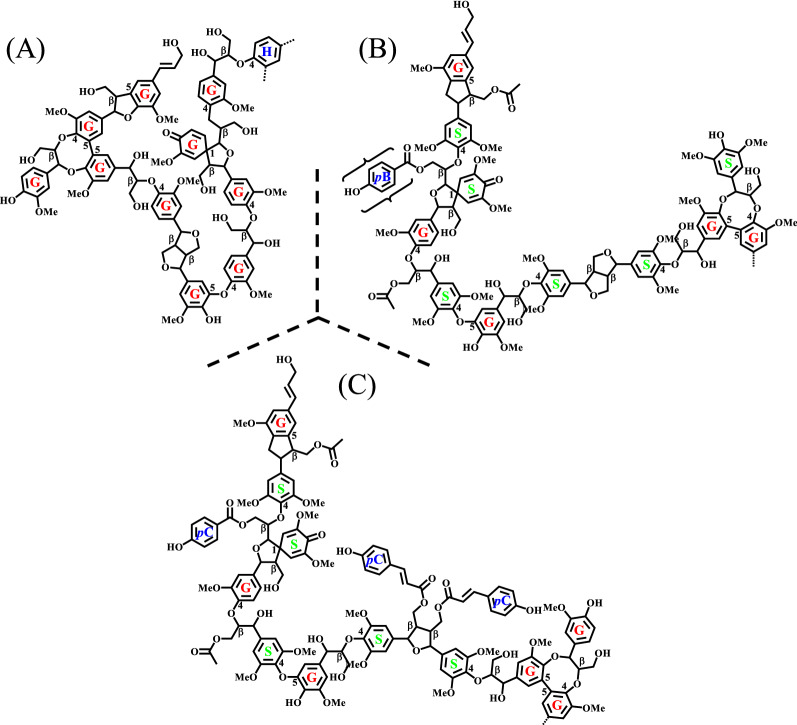
Model lignin structures containing 11 units for **A** a softwood, **B** hardwood, and **C** grass [1]

Due to the natural aromatic polystructure, lignin (which can mainly be extracted from softwood, hardwood, and grass, and the model lignin structures is shown in Fig. 1 [1]) has been regarded as a sustainable alternative to fossil-derived resources to provide valuable biochemicals [2–7]. Interunits of lignin are connected by C–O and C–C bonds (such as β-O-4, β–β, and β-5, Fig. 1), wherein the later contains higher bond dissociation energies (BDE = 69–123 kcal/mol) than that of the former (BDE = 52–80 kcal/mol) [8]. Furthermore, C–C bonds in lignin mainly formed between bulky aromatic rings, making their cleavage more difficult. Besides, C–O bonds are the most abundant linkages in native lignin, accounting for two-thirds or more of total linkages [9–12]. Therefore, much attention has been paid to the efficient cleavage of C–O and C–C bonds in lignin, especially under mild conditions, such as catalytic [13–18], photocatalytic [19–21], ionic liquid promoted depolymerization [22–26], and reductive catalytic fractionation [27–31] to achieve efficient lignin transformation or selective conversion of lignin model compounds to target products. In these studies, some obvious defects can limit their wide applications in the industrial scale, such as the common utilization of noble metal (e.g., ruthenium and rhodium) supported catalysts and lignin model compounds (e.g., dimers containing C–O bonds or lignin-derived monophenols) with high price, non-noble metal supported catalysts with complex preparation processes (such as Ni/Al_2_O_3_ prepared from NiAl-LDH (layered double hydroxide), and Co/MoS_2−x_ with defect sites), and low energy conversion efficiency (e.g., photocatalytic conversion).

Commonly, strategies for lignin conversion to biofuels or biochemicals can be summarized into pyrolysis (thermal and catalytic pyrolysis), hydroprocessing (hydrogenolysis, hydrodeoxygenation, hydrogenation, and integrated hydrogen processing), oxidation (organometallic catalysis, metal-free organocatalytic system, base and acid catalysts, metal salt catalysis, photocatalytic oxidation, and electrocatalytic oxidation), gasification and liquid-phase or steam reforming [9, 32, 33]. Recently, catalytic self-transfer hydrogenolysis has been found as a sustainable method for lignin hydrogenolysis due to the renewable H_2_ source produced from lignin structure [34]. Among these conversion methods, pyrolysis technology has been confirmed as a more effective method in the absence of oxygen due to its easier operation and lower cost for lignin conversion to gases (containing H_2_O, CO, CO_2_, CH_4_, and H_2_), liquid biofuels (containing monophenols or polyphenols), and solid char with shorter reaction time, of which the performance mainly depends on the reaction temperature, time, heating rate and reactor configuration [35–37]. Massive studies have focused on lignin pyrolysis using different techniques such as TGA (thermogravimetric analysis), Py-GC/MS (pyrolysis-gas chromatograph/mass spectroscopy) and fixed-/fluidized-bed reactors, and the products evolution was investigated comprehensively [38–41]. For deeply understanding the pyrolysis mechanism, some studies also used lignin-derived model compounds as pyrolyzed subjects, revealing that several linkages cleavage (such as C_β_–O and C_α_–C_β_) occurred simultaneously [42, 43].

Although lignin pyrolysis mechanism has been explored and lignin oils (rich in phenolic compounds) can be produced, due to the high oxygen content, acidity, and corrosiveness, further upgrading of liquid oils is necessary [44]. Recently, catalytic pyrolysis has been applied as an efficient method for improving oil quality and stability via deoxygenation reactions with assistance of suitable catalysts [45, 46]. The most used catalysts are zeolites, such as ZSM-5, MCM-41, beta, mordenite, ferrierite, and USY with low cost and vast availability, of which the catalytic effect would promote deoxygenation via dehydration, decarbonylation, and decarboxylation reactions [47–49]. Furthermore, functionalities of zeolites on acidity, mesoporosity, and large external surface area make positive effects on aromatics production and char inhibition [50–52]. Aromatic hydrocarbons (AHs), such as monocyclic aromatic hydrocarbons (MAHs) and polycyclic aromatic hydrocarbons (PAHs), are the major products from lignin catalytic pyrolysis using zeolites, wherein naphthalene and anthracene obtained from PAHs can be directly used as fuel additives [53, 54]. On the other hand, for improving oil quality, it is feasible to increase the effective hydrogen index (EHI) of pyrolyzed feedstock due to the uncompetitive content of effective hydrogen in lignin (only around 0.3) [55]. Therefore, lignin co-pyrolysis with other hydrogen-rich feedstock has been proposed as a useful method to improve oil quality [56]. For example, lignin co-pyrolysis with low-density polyethylene over ZSM-5 [57], with polyethylene terephthalate over calcium oxide and zeolites [58], or with plastics (such as polyethylene, polypropylene and polystyrene) over LOSA-1, spent FCC, and Gamma-Al_2_O_3_ [59] showed the enhanced production of AHs owing to the synergistic effect of deoxygenation (from catalysts) and hydrogen transferring (from hydrogen-rich co-feedstock) [60]. In addition, waste cooking oils have been used as hydrogen donors during lignin pyrolysis [61], showing the promising application of co-pyrolysis with some hydrogen-rich feedstock in lignin oil upgrading, which is also feasible for both lignin valorization and other wastes with high value-added recycling.

In this review, we comprehensively summarized the mechanism of lignin pyrolysis (detailedly divided into three stages, e.g., initial, primary and charring stages, and the characteristic products evolution, and mechanism during each stage was introduced, including gas, liquid, and solid). On the other hand, the pyrolytic behaviors of different kinds of lignin (e.g., Kraft lignin, alkali lignin, organosolv lignin, DES lignin, biological lignin, and ionic liquid lignin, also including their isolations, pretreatments, structures, and pyrolytic oil properties) were also introduced in this review. Furthermore, lignin catalytic pyrolysis using zeolites as the most used catalysts and co-pyrolysis with other hydrogen-rich feedstock were also included in this review for improving lignin oils quality via deoxygenation and increasing AHs content, and the corresponding products yield and selectivity were also obtained.

## Lignocellulosic biomass pyrolysis

### Background

Lignocellulosic biomass is a narrow concept of biomass materials, broadly including forestry/agricultural residues, industrial waste biomass, and grasses [62, 63], which is the most popular material for preparing biofuels [64, 65]. Pyrolysis is a kind of thermochemical reaction performed in the absence of oxygen with a certain of heating rate [66], which is an efficient method for converting biomass materials into biofuels [67–71]. Typically, lignocellulosic biomass conversion via pyrolysis technology involves the thermal decomposition of three main basic components in lignocellulosic biomass (i.e., cellulose, hemicellulose, and lignin) [72], which can be transformed into valuable products, such as sugars, cyclopentenes, furans, phenols, and aliphatics in biofuels [73].

### Pyrolysis of cellulose, hemicellulose, and lignin

**Fig. 2:**
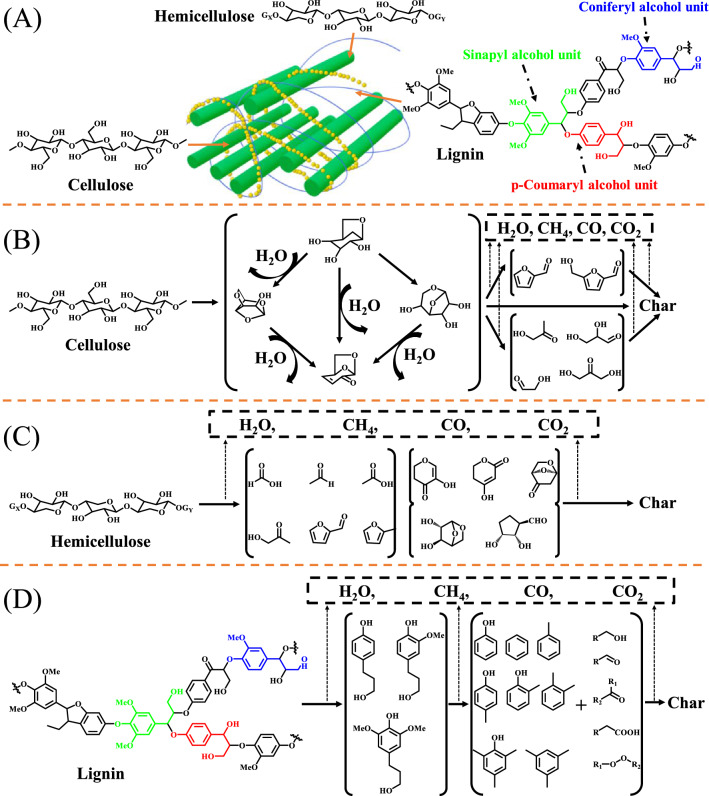
**A** Composition and structure of lignocellulosic biomass, and possible pyrolysis mechanism of **B** cellulose, **C** hemicellulose, and **D** lignin (Reproduced from Refs. [63, 75] with permission from Elsevier)

From Fig. 2A, cellulose is the most abundant polysaccharide in nature, which is a linear polymer of d-glucosyl surrounded by the heterogeneous polysaccharide, hemicellulose, and lignin, and connected by β-1,4-glycoside and intra/intermolecular hydrogen bonds [74]. Due to its relatively simple structure, cellulose has been widely applied in pyrolysis process, briefly including the starting depolymerization at around 350 °C, and further conversion into active cellulosic species, levoglucosan (LGA), levoglucone (LGO), and other monosaccharides via bonds cracking and dehydration. The primary pyrolytic products from cellulose are anhydrosugars, which can be further converted into some light oxygenates (e.g., furans, aldehydes, ketones, acids, etc.) at higher temperatures (Fig. 2B) [75, 76]. In comparison with cellulosic components, hemicellulose has a more complex structure with abundant branched chains and various substituents, which is mainly composed of xylan and glucomannans, where the later contains the similar pyrolytic properties to anhydrosugars (which leads to the similar pyrolytic characteristics to cellulose). Therefore, the pyrolysis process of xylan can be selected as a modifier to study the pyrolytic mechanism of hemicellulose, majorly including initial depolymerization, the generation of furan, and pyran derivatives via dehydration, and the breakage of furanose and pyranose-derived rings to form some light oxygenates (Fig. 2C) [77]. Lignin, a complex biopolymer with aromatic structure, mainly associates with cellulose and hemicellulose together to form plants skeleton. In the process of lignin pyrolysis, higher temperatures are required for linkages cleavage to generate large amounts of phenolic compounds together with small amounts of acid, alcohols, and light aromatic hydrocarbons (Fig. 2D) [78].

### Influencing factors of lignocellulosic pyrolysis

Zhao et al. used commercial cellulose, hemicellulose, and lignin as raw materials without any pretreatments and found that the pyrolytic characteristics can be significantly influenced by the pyrolytic temperature [79]. Besides, different types of lignocellulosic biomass determine various compositions of cellulose, hemicellulose, and lignin, which also influence the pyrolytic characteristics. Typically, cellulosic components can produce the higher yield of biofuels (~ 60%) compared to those of hemicellulose (~ 40%) and lignin (~ 30%). Other influencing parameters, such as the contents of ash, metal, and moisture, also affect the production and properties of final generated biofuels. Higher contents of moisture in lignocellulosic biomass would reduce the calorific value and deteriorate the quality of biofuels.

During pyrolysis, the function of pyrolytic temperature is to provide heat to decompose the structure of substrates, and increasing the pyrolytic temperature can enhance the conversion efficiency. At lower pyrolytic temperatures, ash in raw materials would be retained in solid products to yield more semi-coke. In addition, metals existing in the ash, such as kalium and calcium, can enhance the pyrolytic heating rate while reduce the yield of biofuels due to their catalyzed effect to promote the secondary cracking reactions among products in biofuels [80]. For example, Tsai et al. used rice husk as raw material with further grinding and rose the pyrolytic temperature from 400 to 500 °C, leading to the higher production of biofuels from 11.26 to 35.92 wt% and reaching the maximum yield (i.e., 38.8 wt%) at 800 °C [81]. Lazzari et al. used mango fruit seeds as raw materials (which were separated into tegument and almond) and obtained the highest yield of biofuels at 38.8 wt% when the pyrolytic temperature increasing to 650 °C [82]. It is suggested that increasing the pyrolytic temperature makes a positive effect on the production of biofuels, while the opposite trend would be found at a very high temperature due to the occurrence of the secondary cracking reactions [83]. Jung et al. used rice straw and bamboo sawdust as raw materials with further sieving into small particle sizes and pointed out that as the pyrolytic temperature of bamboo sawdust increases over 500 °C, biofuel yield would gradually decrease, and the optimal pyrolytic temperature for rice straw is 445 °C to yield more biofuels [84]. In addition, the optimal pyrolytic temperature ranges for rice husks/sawdust and cotton stalk without ant pretreatments are confirmed at 420–540 and 480–530 °C, respectively [85–87], and the maximum yield of biofuels for rice husk (with further grinding) (70.0 wt%) [88], palm waste (with further grinding) (72.4 wt%) [89], neem de-oiled cake (without any pretreatments) (40.2 wt%) [90], *Cynara cardunculus* (without any pretreatments) (56.2 wt%) [91], olive bagasse (without any pretreatments) (46.3 wt%) [92], sugarcane bagasse (with further drying, grinding, and sieving) (61.4 wt%) [93], cassava rhizome (with further drying, grinding and sieving) (63.2 wt%) and cassava stalk (with further drying, grinding and sieving) (61.4 wt%) [94], Jatropha seed shell cake (without any pretreatments) (48.0 wt%) [95], pistachio shell (with further drying and grinding) (20.5 wt%) [96], and polar wood (without any pretreatments) (69.0 wt%) [97] can be obtained at 450, 500, 400, 400, 600, 475, 472, 469, 470, 550, and 455 °C, respectively. Biswas and Pattiya et al. also suggested that different kinds of biomass materials (e.g., corn cob, wheat straw, rice straw and rice husk, with further drying, crushing and sieving) contain their own ideal pyrolytic temperatures to generate the highest yield of biofuels [98, 99].

Except for the pyrolytic temperature, Qiu et al. pointed out that the type of biomass feedstock is also an important factor to influence the generation of biofuels due to the differences of their compositions and structures [72]. Oasmaa et al. found that wood biomass can produce more biofuels with lower yield of aqueous and gaseous products [100]. Among these formed biofuels, different compositions of raw materials lead to the different properties of biofuels. For example, cellulosic-derived biofuels are rich in acids, furans, and ketones, which are similar to those of hemicellulose-derived ones, while lignin-derived biofuels reveal a great capacity to produce phenolic compounds [101]. There is a positive correlation of ketones and cellulose, furans and holocellulose, and phenols and lignin, while there is a negative correlation of short-chain acids and ash, and hydrocarbons and cellulose. Furthermore, with increasing the pyrolytic temperature, various interactions occur to change the composition of biofuels. For example, Huang et al. found that alkenes, alkanes, long-chain fatty acids/esters, aliphatic nitriles, and amides can be obtained at lower pyrolytic temperatures, while higher pyrolytic temperatures favor the cracking reaction among aliphatic species and the formation of aromatic compounds [102]. Ly et al. found that a higher pyrolytic temperature produces biofuels with more short aliphatic carbons, low-molecular-weight alcohols, ketones, and their derivatives due to the further decomposition reaction occurring [103]. Alvarez et al. observed that increasing the pyrolytic temperature would reduce the yield of ketones and acids, while increase the yield of phenols [88]. In addition, the pyrolytic temperature is also an important factor for determining the quality of biofuels, such as H/C ratio, which is an essential indicator for HHV (high heating value, kJ/mol) [104]. Ates and Ly et al. also observed that increasing the pyrolytic temperature is beneficial for reducing oxygen content in biofuels, while increasing the content of hydrogen, showing the higher calorific value of biofuels [105, 106].

### The meaning of further investigations on lignin pyrolysis

Even though massive studies have focused on lignocellulosic biomass pyrolysis, the biggest challenge is about the high-efficiency conversion of lignin. Lignin accounting for 40% of energy (higher than that of polysaccharides) has been considered as the most promising source for biochemical platform and bioenergy due to its aromatic structure [107, 108]. Nowadays, over 90% of lignin is burned with low efficiency, and only 2% of lignin is utilized efficiently due to its complex structure and low reactivity [109, 110]. As the stubbornest component in lignocellulosic biomass, the efficient conversion of lignin has attracted much attentions from both industry and research community and is still a researching hotspot up to now. Pyrolysis technology has been considered as an effective method for lignin conversion to well-fined chemicals due to its low cost, easy operation, high efficiency for energy extraction, and fuel production [111–114]. As the only renewable alternative of non-renewable fossil fuels for producing aromatic compounds, lignin can be thermally decomposed via pyrolysis technology to obtain low-molecular-weight phenolic compounds (important intermediates in biochemical platform) and to generate aromatic hydrocarbons (advanced fuel additives with sustainable energy density) [115]. On the other hand, biorefinery economic can benefit from high-quality recovery of lignin by lowering the manufacturing price of biochemicals and biofuels [116].

## Lignin pyrolysis

### Background

Lignin is one of the three main components in lignocellulosic biomass, combined with cellulose and hemicellulose via hydrogen and ester bonds [117–119]. So far, large quantities of lignin have been produced from industrial and agricultural activities, and pyrolysis technology has been considered as an effective method for its high-efficiency conversion [120–122]. Unfortunately, the presence of cellulose and hemicellulose would affect the pyrolytic behavior of lignin owing to a series of coupling interactions occurring [123–125], hence leading to a more intangible pyrolysis process. After delignification, different kinds of lignin with high purity and homogeneity can be extracted from raw biomass and then pyrolyzed into low-molecular-weight compounds, which are the so-called pyrolytic oils (mainly composed of phenolic compounds, aldehydes, acids, and other aromatic hydrocarbons) [126, 127].

### Lignin pyrolysis mechanism

Typically, the radical reaction of lignin pyrolysis has been established and divided into three stages: initial, primary, and charring stages (or namely the radical inducing, the main reacting and the quenching stages [126]). Prior to the detail description of their own pyrolytic characteristics and product distributions, we first introduce the general pyrolytic mechanisms of lignin pyrolysis.

#### General pyrolytic mechanism of lignin

**Fig. 3:**
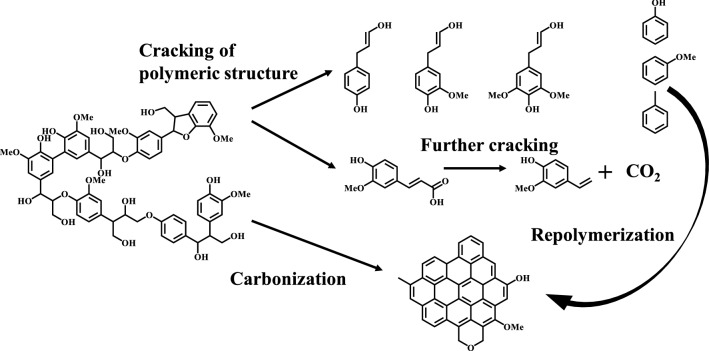
Proposed pyrolytic mechanism of lignin (Reproduced from Ref. [128] with permission from Elsevier)

Lignin pyrolytic mechanisms can be researched via several techniques, such as thermogravimetric analysis (TG), Fourier transform infrared spectroscopy (FTIR), and gas chromatography/mass spectrometry (GC/MS) to determine mass loss, volatiles evolution, and decomposed products distribution, respectively. For example, a coupling analysis of TG-FTIR-GC/MS was used by Yang et al. to investigate the pyrolytic mechanism of lignin-rich residue obtained from *Arundo donax*. Pyrolytic results including the composition, carbon number and atomic ratio (C/H, C/O) of bio-oils was obtained for proposing pyrolysis mechanism and possible reaction pathway [128]. Through TG analysis, three main stages of lignin pyrolysis can be obtained, including water evaporation (< 200 °C), primary decomposition (at 250–500 °C), and carbonization (> 500 °C) [129–132]. Through the coupling analysis of TG, FTIR, and GC/MS, lignin pyrolytic mechanism can be elucidated in Fig. 3, where chemical linkages, such as C–C and C–O–C bonds, can be cleaved to produce phenols and aromatic hydrocarbons, and functional groups, such as carboxyl, carbonyl, and methoxy, would be escaped from benzene ring to form some light gases. Finally, lignin biochar with abundant functional groups (such as –OH and –OCH_3_) would be generated via carbonization [133]. On one hand, functional groups in biochar would be further cracked with the temperature rising to form light gases. On the other hand, active aromatic species in bio-oils would be transformed into biochar via repolymerization.

#### Influencing factors of lignin pyrolysis

Yu et al. investigated the importance of pyrolytic temperature on lignin pyrolysis, where monophenols are the main components at the temperature range of 450–525 °C, and higher temperatures prefer the generation of dimers [134]. Through the further cracking reactions of carboxylic acid, ketone, and aldehyde, and the inhibition of coupling reactions of free mono radicals, the production of phenols can be enhanced. Besides, some functional groups in lignin, such as methoxy and phenolic hydroxyls, significantly change the pyrolytic characteristics of lignin [135]. Methoxy is an important functional group in lignin, which can affect the repolymerization degree of lignin during pyrolysis [136], and also is defined as an important factor to promote the formation of char residues [137–139]. The positive effect of demethylation on repolymerization inhibition can be confirmed by the lower char yield of demethylated lignin compared to that of raw lignin. Besides, with the enhancement of demethylation degree, the main components of demethylated lignin pyrolytic products are adjusted from methoxyphenols to H-type phenols, such as phenols, methyl phenols, and catechol [140]. On the other hand, the charring mechanism of lignin pyrolysis suggests that (1) char cannot be generated when there is only one phenolic hydroxyl; (2) a bromine group as an electron withdrawing site can decrease electron cloud density in *ortho* position, reduce the bond dissociation energy of phenolic hydroxyl, and promote dehydrogenation, which is conductive for char formation; (3) methoxy, as an electron-donating group, does not promote the repolymerization reaction, while phenolic hydroxyl, as an electron donor group, can promote repolymerization by affecting electron could density; (4) the intramolecular reaction between methoxy and phenolic hydroxyl groups significantly promote the repolymerization, and the promotion of char formation is directly proportional to the amount of methoxy in the *ortho* position of phenolic hydroxyl [140].

#### Initial stage of lignin pyrolysis

**Fig. 4:**
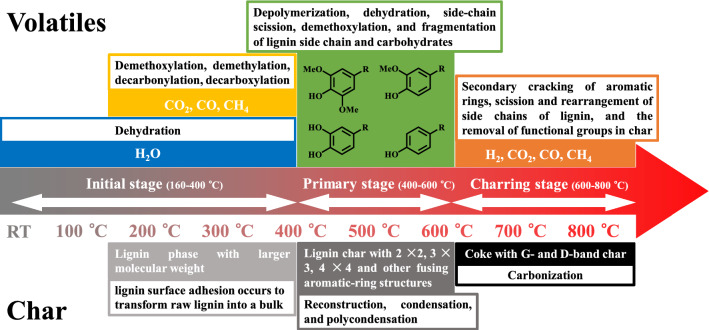
Characteristic pyrolytic behaviors of initial, primary, and charring stages

Lignin pyrolysis process can be divided into three stages, including initial, primary, and charring stages, and each stage has its own characteristic pyrolytic behaviors (Fig. 4) and mechanisms (introduced in the following sections).

Li et al. investigate the reaction pathway of initial stage comprehensively, revealing that a complex initial reaction of lignin pyrolysis mainly occurs at the temperature range of 160–330 °C (before 160 °C, external and internal water removing causes a light mass decrease) with mass loss of approximately 20% to form considerable amounts of volatile products [141]. During initial stage, some light gaseous products start to release (e.g., H_2_O releasing at the ranges of 160–330 and 330–400 °C due to dehydroxylation and hydroxyl cracking in lignin hydrogen bond network; CO_2_ releasing at the ranges of 160–270, 270–330, and 330–400 °C due to fatty ether bonds cleavage and oxidation, carboxylic acid group cleavage, and carbonyl group cleavage and oxidation; CO releasing at the ranges of 170–330 and 330–400 °C due to the cleavage of ether bonds and side chains C–O; CH_4_ releasing at the range of 330–400 °C due to the methoxy cleavage). The variations of activation energy of these light gases are mainly due to their formations from different sources with different bond energies [142]. When the temperature increases up to 150 °C, the polymerization reaction happens to form the condensed lignin phase with a larger molecular weight, and oil-based components (such as lignin monomers, dimers, and trimers) would be orderly generated after the temperature further rising to 330 °C [141]. On the other hand, lignin surface adhesion occurs to transform raw lignin into a bulk, and the evolution of functional groups illustrates that hydroxyl and hydrocarbon groups in bulk lignin are temperature induced (of which the content decreases as the temperature increases to form more light gases). In addition, non-conjugated carbonyl, conjugated carbonyl, aromatic, guaiacyl, and syringyl groups in lignin are all temperature induced, which can be converted into aromatic compounds, together with the escape of side chains to form light gases with increasing pyrolytic temperature. Finally, the structure polymerization of condensed lignin or the repolymerization among aromatic compounds happen to generate biochar with fused aromatic rings.

The structural changes of char produced from initial stage was investigated by Chua et al. [143]. With increasing temperature (over the softening temperature of lignin at ~ 140 °C [144]), the content of THF-insoluble fraction of obtained lignin char contains a larger molecular weight, indicating the occurrence of repolymerization reaction. For THF-soluble fraction, almost the half is transformed into volatile products and the remainder is repolymerized into THF-insoluble fraction (of which the structure is composed of mono, 2–3 fused, and 3–5 fused aromatic rings [145]) when the temperature increases up to 300 °C. From RT to 250 °C, the aromatic structure of char is reduced due to the generation of light gases and oil component compounds (e.g., phenolic monomers, dimers or trimers). The decrease of both H/C and O/C ratios in THF-soluble fraction with increasing temperature from 100 to 300 °C illustrates that some mono-aromatics would be repolymerized into condensed phases with fused-ring structures in THF-insoluble fraction. The change of char structure with the increase of temperature illustrates that (1) the aliphatic carbon structure decreases due to the escape of alkyl side chains, (2) the methoxy carbon structure decreases due to the decomposition of ether bonds (such as α-O-4 and β-O-4), (3) the aromatic carbon structure increases due to the enhanced aromaticity, and (4) the carbonyl carbon structure decreases due to the release of light gases (such as CO_2_).

#### Primary stage of lignin pyrolysis

Primary stage of lignin pyrolysis containing more intense structural decomposition happens at the temperature range of 400–600 °C after initial stage, where phenolic compounds (which is both temperature induced and time induced) are the major components during this stage [146–148]. The pyrolytic temperature and residence time have significant influences on products generation [149–151]. Lin et al. investigated the effect of temperature and time on the generation of phenolic products, illustrating that the maximum yield of phenolic products can be obtained at 450 °C [152]. With increasing temperature, the production of phenolic monomers is enhanced significantly due to the increasing conversion rate of lignin [153–155]. However, due to the secondary reaction occurring with the extension of reaction time, more phenolic monomers would be transformed into light gases (which leads to no further increase of the detecting response of phenolic monomers at 600 °C when the residence time increases from 1 to 5 s). On the other hand, the reaction equilibrium would also lead to no obvious enhancement of products generation at 600 °C with the extension of residence time [127, 155]. Expect for the temperature and reaction time, lignin structure also influences the pyrolytic behavior at primary stage [155, 156]. For example, Marathe et al. investigated the effect of molecular weight on lignin pyrolysis, and the results demonstrate that (1) the increasing molecular weight of lignin would decrease the oil yield due to higher yields of gases and solids obtained; (2) the molecular weight of liquid products is independent to that of lignin but is associated with the reaction pressure; and (3) the yield of liquid products is proportional to the reaction temperature [157]. Besides, Yuan et al. investigated the effect of lignin structure on the formation of phenolic compounds, and various lignin extracted from softwood (pine), hardwood (eucalyptus), and herbaceous feedstock (corn stalk and bamboo) were pyrolyzed, presenting different compositions of liquid products, wherein lignin samples from corn stalk and bamboo favor H-type phenols, and that from pine prefers G-type phenols, while that from eucalyptus majorly produces polyphenols [158].

**Fig. 5:**
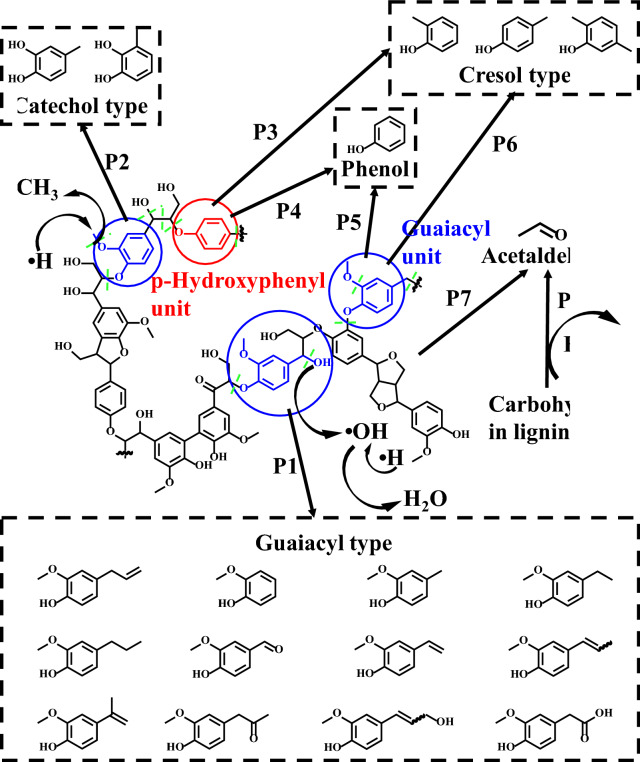
Proposed reaction pathways for primary stage of lignin pyrolysis (**P1**: depolymerization and dehydration; **P2**: side-chain scission and demethylation; **P3**, **P4**: depolymerization; **P5**: demethoxylation; **P6**: side-chain scission and demethoxylation; **P7**: fragmentation of lignin side chains; **P8**: dehydration and fragmentation of carbohydrate) [39]

At the temperature range of 400–600 °C, the structure decomposition of lignin is driven by the linkage cleavage (e.g., ether bonds with bond dissociation energy (BDE) of ~ 291 kJ/mol, and C–C bonds with BDE of ~ 323 kJ/mol), wherein the chemical linkage with lower BDE would be broken easier with the increase of temperature, while that with higher BDE would be more temperature resistance, which can be presented as functional groups in final products [159–161]. In the process of linkages cleavage, hydroxyl radicals are produced from scission of aliphatic side chains, and hydrogen radicals can be generated from β-scission reactions (from C–OH to C=O), which might migrate to either aliphatic side chains or aromatic rings with oxygen radicals [162, 163]. Furthermore, due to the high temperature resistance property of methoxy (i.e., BDE = 481 kJ/mol), large amounts of methoxylated phenols are generated [9, 164]. From Fig. 5, the obtained products can be mainly categorized as guaiacyl, cresol, and catechol types, where guaiacyl units are suggested to produce G-type phenols via depolymerization and dehydration (**P1**) [165]. Cresol and catechol are generated by the cleavage of interunit linkages and the cracking of aliphatic side chains (**P2**, **P3,** and **P6**). The higher temperature would lead to more demethylation and demethoxylation reactions of guaiacyl units (**P2** and **P6**) to increase the yield of cresol and catechol-type compounds [160, 166]. In addition, cresol-type compounds can also be generated from *p*-hydroxyphenyl units via depolymerization (**P3** and **P4**) [167]. Phenol-type compounds are created by demethoxylation (**P5**) of guaiacyl units [168], and acetaldehyde is suggested to be formed from carbohydrate fractions and side chains via dehydration (**P8**) and fragmentation (**P7**), respectively [77, 149, 169]. In addition, water can be produced via dehydration reaction between hydroxyl and hydrogen radicals, and methanol and methane can be generated from methyl radicals via demethoxylation promoted by hydrogen or hydroxyl radicals [163].

Yang et al. elucidated lignin pyrolysis at primary stage comprehensively (i.e., from 200 to 600 °C), especially for the structural evolution of char at this stage [170]. After 400 °C, H_2_ has been gradually generated and obtains its maximum content at 600 °C due to the polymerization of aromatic rings [127]. With the increase of temperature (from 350 to 600 °C), the condensation of aromatic rings happens to form char residues with polycondensed aromatic structure. The evolution of char structure includes the conversion of hydroxyl groups to carbonyls and aliphatic chain C–H to alkene C–C, mainly due to the primary structure decomposition, and aromatic condensation reactions occurring to cause the aromatic polycondensation over 400 °C. The pyrolysis mechanism can be summarized as (1) almost no lignin conversion happens before 200 °C due to its thermal stability, (2) aromatic side chains can be cleaved to form some light gases as the temperature increasing up to 250 °C [171], (3) from 250 to 350 °C, depolymerization and condensation exist as competing reactions to generate abundant liquid products (i.e., methoxyphenols) via linkages cleavage [172], (4) from 350 to 450 °C, the condensation reaction is enhanced, including repolymerization and condensation, to generate char with 1 × 2 and 2 × 2 fused-ring structures, (5) at 450–600 °C, demethylation, demethoxylation, and dehydrogenation reactions occur to form CH_4_ and H_2_, and further convert guaiacyl-type compounds into catechol-type ones and alkyl phenols [173], and (6) at 450–600 °C, the structure of lignin char can be generated consisting of 2 × 2, 3 × 3, 4 × 4, and other fusing rings.

#### Charring stage of lignin pyrolysis

**Fig. 6:**
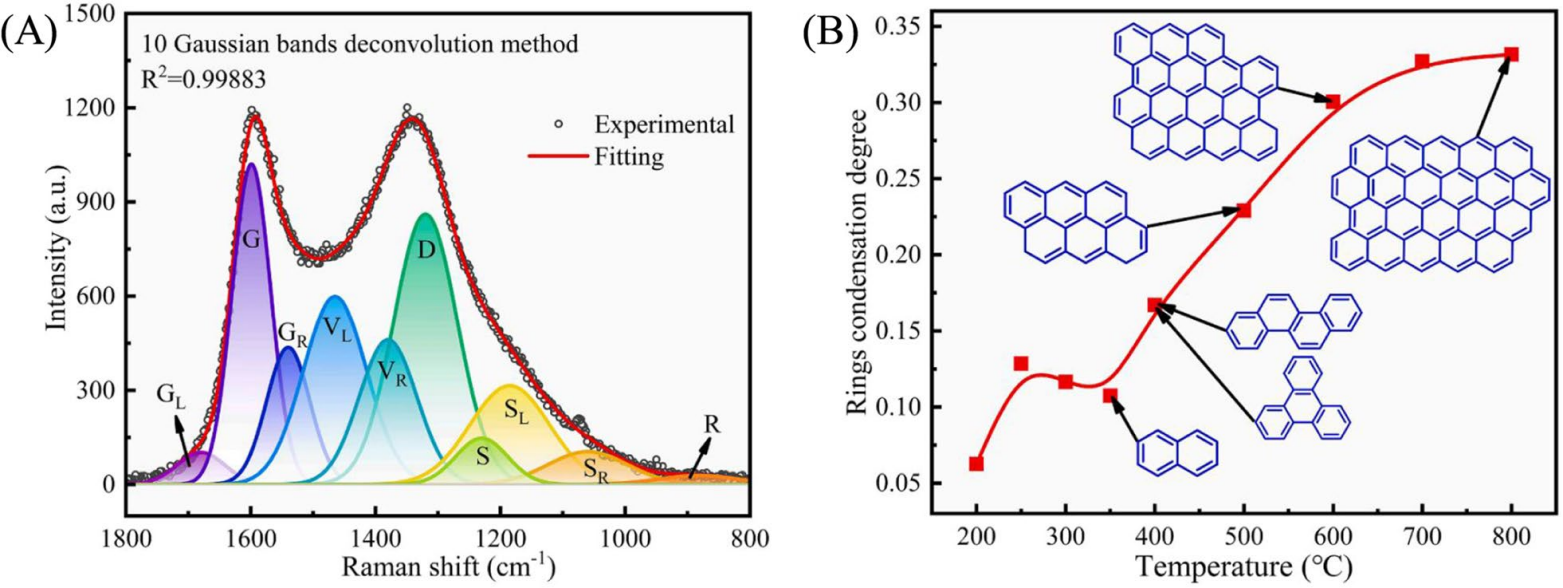
**A** Curve fitting for the first-order Raman spectrum of char obtained at 800 °C, and **B** relationships between ring condensation degree and pyrolytic temperature (Reproduced from Ref. [40] with permission from Elsevier)

Through the above studies on lignin pyrolysis, it is well known that the structure of lignin char changes obviously with increasing temperature. Zheng et al. studied the char structure evolution during lignin fast pyrolysis from 200 to 800 °C [40]. Raman spectra in Fig. 6A show that the graphitization degree of lignin char is relatively low due to the presence of low-strength G band of graphite crystals (~ 1585 cm^−1^) [174]. Figure 6A shows the fitting curves of Raman spectrum of char obtained at 800 °C, consisting of G_L_ (at 1680 cm^−1^, carbonyl group C=O), G (at 1585 cm^−1^, graphite E^2^_2g_; aromatic ring quadrant breathing; alkene C=C), G_R_ (at 1540 cm^−1^, aromatics with 3–5 rings; amorphous carbon structures), V_L_ (at 1465 cm^−1^, methylene or methyl; semi-circle breathing of aromatic rings; amorphous carbon structures), V_R_ (at 1380 cm^−1^, methyl group; semicircle breathing of aromatic rings; amorphous carbon structures), D (at 1320 cm^−1^, D band on highly ordered carbonaceous materials; C–C between aromatic rings and aromatics with not less than 6 rings), S_L_ (at 1230 cm^−1^, aryl–alkyl ether; *para*-aromatics), S (at 1185 cm^−1^, C_aromatic_–C_alkyl_; aromatic (aliphatic) ethers; C–C on hydro-aromatic rings; hexagonal diamond carbon sp^3^; C–H on aromatic rings), S_R_ (at 1060 cm^−1^, C–H on aromatic rings; benzene (*ortho*-di-substituted) ring), and R (at 960–800 cm^−1^, C–C on alkanes and cyclic alkanes; C–H on aromatic rings) [174, 175]. Raman bands of inorganic matters are mostly below 1100 cm^−1^; therefore, organic components occupy the main proportion of lignin char. Figure 6B shows the relationship of ring condensation degree versus temperature, elucidating that the aromatization starts at 350 °C, followed by the evolution of char structure from 2 × 3 fused ring to 4 × 5 fused ring over 700 °C. The behavior of carbon deposit on fusing rings would be enhanced owing to the recombination and recondensation of volatile products at higher temperatures [176]. Besides, not only temperature but also heating rate can affect the formation and structure of char significantly. Li et al. investigated the effect of heating rate on the evolution of functional groups and structural properties of lignin char, indicating that the higher heating rate would increase the oxygen content and decrease the HHV value of char, and lead to the further structural decomposition [38].

### Recent progress of lignin pyrolysis

According to the pyrolysis mechanism presented above, in this section, we mainly introduce the pyrolytic behaviors of different types of lignin, including Kraft lignin (Table 1), alkali lignin (Table 1), organosolv lignin (Table 1), DES (deep eutectic solvents) lignin (Table 2), biological lignin (Table 3), and ionic liquid lignin (Table 4), and their own characteristic reaction pathways.

**Table 1:** Structures, isolations, and/or treatments of Kraft lignin, organosolv lignin, and alkali lignin, and their pyrolytic behaviors

Raw material	Isolation	Treatment	Lignin	Lignin properties	Pyrolysis temperature	Pyrolytic property		Refs.
						Yield	Major compositions in oils	
Commercial Kraft lignin (KL)	N/A	N/A	KL	G/S-type lignin with C = 62.45%, H = 5.28%, N = 0.27%, O = 31.01%, S = 0.16%, ash = 0.83, H/C_eff_ = 0.63	RT-400–700 °C^a^	400 °C^a^—gas/oil/char (10/21/69%); 500 °C^a^—gas/oil/char (19/33/48%); 600 °C^a^—gas/oil/char (24/34/43%); 700 °C^a^—gas/oil/char (33/29/38%)	400–700 °C^b^ (S-type phenols from 58 to 72%, G-type phenols from 18 to 22%)	[188]
Norway spruce (*Picea abies*)	LignoBoost process	N/A	KL (Lignoboost™ lignin)	G-type lignin with HHV (dry ash free) = 27.1 MJ/kg, HHV (moist) = 18.2 MJ/kg, C = 65.1%, H = 5.8%, N = 0.1%, O = 26.1%, S = 2.5%	RT-500 °C^c^	500 °C^c^—char (36%)	500 °C^c^ (guaiacols, catechols, alkylphenols)	[116]
Black liquor of eucalypt Kraft pulping	Acid precipitation	Thermal treatment (175, 200, 225 °C for 30, 90, 150 s)	Thermal-treated KL	KL-200 °C (90 s) (exp.): G/H/S-type lignin with C = 66.30%, H = 5.63%, N = 0.11%, O = 26.07%, S = 1.70%, HHV = 26.52 MJ/kg, soluble lignin = 5.97%, insoluble lignin = 91.41%, sugars = 0.80%, ash = 1.56%	RT-550 °C^c^	550 °C^d^—char (~ 10%)	225 °C^c^ (methoxycatechol syringol, 4-methylsyringol)	[189]
Black liquor of poplar Kraft pulping	Acid precipitation	Fractionation by ultrafiltration	KL and F1 (54.3%), F2 (25.9%), F3 (15.4%), F4 (4.4%) fractionated in series by membranes with 10, 5, 1 kDa	KL/F1/F2/F3/F4: G/H/S-type lignin with *M*_n_ = 584/1141/703/484 g/mol, *M*_w_ = 1507/2640/1119/700 g/mol, *Ð*^e^ = 2.58/2.31/1.59/1.44, acid-insoluble lignin (AIL) = 76.68/78.41/77.62/75.35/76.56%, acid soluble lignin (ASL) = 5.19/5.31/7.09/7.13/6.97%, total carbohydrates = 4.99/8.11/3.69/0.07/0%, ash = 0.95/1.84/1.19/1.07/1.09%	RT-500, 650, 800 °C^c^	750 °C^d^—char: KL (35.1%), F1 (33.8%), F2 (25.3%), F3 (31.8%)	500 °C^c^: KL, F1, F2, F3 (syringol, 4-methylguaiacol, guaiacol); 650 °C^c^: KL (syringol, 4-methylguaiacol, guaiacol), F1 (4-methylguaiacol, guaiacol, 4-vinylguaiacol), F2 (syringol, guaiacol, phenol), F3 (syringol, guaiacol, 4-vinylguaiacol); 800 °C^c^: KL (phenol, syringol, toluene), F1 (phenol, 4-methylphenol, 2,4-dimethylphenol), F2 (syringol, guaiacol, 4-vinylguaiacol), F3 (phenol, syringol, 4-vinylguaiacol)	[154]
Black liquor of eucalypt Kraft pulping	Acid precipitation	Fractionation by organic solvents	KL and F1 (59.65%), F2 (28.70%), F3 (10.93%) fractionated in series using ethyl acetate (F1), acetate/petroleum ether (F2), petroleum ether (F3)	KL/F1/F2/F3: G/S-type lignin with *M*_n_ = 2033/2872/1611/620 g/mol, *M*_w_ = 4664/6158/2397/814 g/mol, *Ð*^e^ = 2.29/2.14/1.49/1.32, C = 61.30/61.01/62.26/62.49%, H = 5.56/5.59/5.80/5.92%; N = 0.19/0.28/0.01/0.00%, O = 30.81/30.86/29.92/29.91%, S = 2.14/2.26/2.01/1.68%, O/C (mol/mol) = 0.38/0.38/0.36/0.36, H/C (mol/mol) = 1.09/1.10/1.12/1.14, HHV = 24.86/24.80/25.55/25.73 kJ/kg, S/G (unit ratio) = 2.44/2.12/2.32/2.49, β-O-4 = 3.7/12.6/2.8/0%, β–β = 1.2/2.2/0.9/0.3%, β-5 = 5.4/5.5/4.8/4.2%, aliphatic OH = 1.06/1.47/0.84/0.29 mmol/g, total phenolic OH = 3.81/2.51/3.49/4.81 mmol/g, COOH = 0.54/0.49/0.52/0.73 mmol/g, condensed phenolic OH = 0.48/0.35/0.54/0.81 mmol/g, methoxy = 6.86/6.11/6.59/7.19 mmol/g	RT-400, 500, 600 °C^f^	600 °C^d^—char: KL (40.61%), F1 (38.88%), F2 (33.32%), F3 (18.37%)	400 °C^b, g^: KL, F1, F2, F3 (G-/S-type phenols); 500 °C^b, g^: KL, F1, F2, F3 (G-/S-type phenols); 600 °C^b, g^: KL, F1, F2, F3 (G-/S-/H-type phenols)	[135]
Black liquor of poplar Kraft pulping	Acid precipitation	Fractionation by gradient acid precipitation	KL (extracted under pH = 2) and L_pH6_ (52.5%), L_pH4_ (29.7%), L_pH2_ (17.8%) fractionated in series under pH = 6, 4, 2	KL: G/S/H-type lignin with *M*_n_ = 821/1057/703/462 g/mol, *M*_w_ = 2118/2399/1057/712 g/mol, *Ð*^e^ = 2.58/2.27/1.73/1.54, C = 52.89/60.31/60.27/48.42%, H = 4.85/5.28/5.28/3.86%, N = 0.15/0.16/0.30/0.22%, O = 29.51/32.57/32.13/23.61%, S = 12.60/1.76/2.02/23.89%	RT-500, 650, 800 °C^c^	750 °C^d^—char: KL (35.1%), L_pH6_ (38.4%), L_pH4_ (29.1%), L_pH2_ (26.8%)	500 °C^c^: KL, L_pH6_, L_pH4_ (syringol, 4-methylguaiacol, guaiacol), L_pH2_: (syringol, 4-ethylguaiacol, guaiacol); 650 °C^c^: KL (syringol, 4-methylguaiacol, guaiacol), L_pH6_, L_pH2_ (syringol, 4-ethylguaiacol, guaiacol), L_pH4_ (3-methoxycatechol, syringol, 4-methylguaiacol); 800 °C^c^: KL (phenol, syringol, toluene), L_pH6_ (acetosyringone, 3-methoxycatechol, phenol), L_pH4_ (syringol, 4-ethylguaiacol, 4-methylguaiacol), L_pH2_ (syringol, phenol, guaiacol)	[190]
Commercial alkali lignin (AL)	N/A	N/A	AL	G/H/S-type lignin with C = 55.3%, H = 5.3%, N = 0.1%, O = 35.2%, S = 1.3%, HHV = 20.62 MJ/kg, volatiles = 62.3%, fixed carbon = 34.9%, ash = 2.8%	RT-400, 450, 500 550, 600, 650, 700, 750, 800 °C^a^	600 °C–char^d^ (39.0%); 800 °C—char^d^ (40.8%); 400–800 °C^a^—gas/oil/char (15/25/65%)	400–800 °C^b^ (phenol, 2-methylphenol, 4-methylphenol, 4-ethylphenol)	[221]
Commercial alkali lignin	N/A	N/A	AL	G/H/S-type lignin with C = 60.00%, H = 7.18%, N = 0.00%, O = 29.90%, S = 2.92%, HHV = 20.62 MJ/kg, moisture = 6.2%, volatiles = 88.0%, fixed carbon = 9.4%, ash = 2.6%	RT-300, 350, 400, 450 °C^a^	300 °C^a^—gas/oil/solid (11.8/26.2/62.0%); 350 °C^a^—gas/oil/solid (12.7/32.3/55.0%); 400 °C^a^—gas/oil/solid (16.5/33.5/50.0%); 450 °C^a^—gas/oil/solid (20.9/34.1/45.0%)	300, 350, 400, 450 °C^b^ (guaiacols, alkyl phenols, catechols)	[204]
Commercial alkali lignin	N/A	N/A	AL	G/H/S-type lignin	40–900 °C^c, i^	900 °C^d^—char (~ 50%)	300, 500, 900 °C^c^ (2-methoxy-phenol)	[222]
Commercial alkali lignin	N/A	N/A	AL	G/H-type lignin with aliphatic OH/COOH/phenolic OH/total OH/H (unit)/G (unit) = 2.36/0.38/3.35/6.09/0.27/3.08 mmol/g	RT-400, 500, 600 °C^c^	N/A	400 °C^b^ (no obvious response); 500, 600 °C^b^ (2-methoxyl-4-methylphenol, acetaldehyde, 2-methoxyl-4-vinylphenol, 2-methoxyl-phenol)	[39]
Black liquor (BL)	Acid precipitation	Fractionation by ultrafiltration	AL, AL-10/5/1 kDa	AL, AL-10/5/1 kDa: G/H-type lignin with *M*_n_ = 3284/6076/4045/2616 g/mol, *M*_w_ = 7423/12,060/6752/3864 g/mol, *Ð*^e^ = 2.260/1.985/1.669/1.478, G-type OH = 0.90/1.07/0.86/0.77 mmol/g, H-type OH = 0.22/0.15/0.17/0.30 mmol/g, aliphatic OH = 1.23/1.47/1.06/1.05 mmol/g, condensed phenolic OH = 1.21/1.74/1.17/1.00 mmol/g, COOH = 1.02/1.19/0.74/0.76 mmol/g	RT-400, 600, 800 °C^h^	1000 °C^d^—char: AL (43.06%), AL-10 kDa (38.42%), AL-5 kDa (39.27%), AL-1 kDa (36.34%)	400 °C^b^: AL, AL-10 kDa (guaiacol, 4-methylguaiacol, 4-ethylguaiacol), AL-5 kDa, AL-1 kDa (guaiacol, phenol, 4-methylguaiacol); 600 °C^b^: AL, AL-10 kDa, AL-5 kDa (guaiacol, phenol, 4-methylguaiacol), AL-1 kDa (phenol, 4-methylphenol, 3-ethylphenol); 800 °C^b^: AL, AL-10 kDa, AL-5 kDa (phenol, indene, naphthalene), AL-1 kDa (1-ethenyl-2-methyl-benzene, indene, naphthalene), (39.27%)	[205]
Corncob residue	Organosolv method (tetrahydrofuran)	N/A	Organosolv lignin (OL)	G/S/H-type lignin with *M*_n_ = 1238 g/mol, *M*_w_ = 3203 g/mol, *Ð*^e^ = 2.59	RT-800 °C^a^	350 °C^a^—gas/oil/char (31.5/16.3/52.2%); 800 °C—char^d^ (36.02%)	350 °C^b^ (4-vinylphenol, 4-vinylguaiacol, syringol)	[223]
Pine, poplar, bamboo	Organosolv method (dioxane)	N/A	PiL (29.3%), PoL (35.2%), BaL (48.9%)	PiL: G/H-type lignin with *M*_n_ = 2580 g/mol, *M*_w_ = 9940 g/mol, *Ð*^e^ = 3.85, β-O-4 (A) = 46.02%, phenylcoumarans (B) = 8.63%, resinol (C) = 5.78%, cinnamyl alcohol end groups (I) = 6.72%, S/G/H (unit) = 0/99.35/0.65%; PoL/BaL: G/S/H-type lignin with *M*_n_ = 3540/3420 g/mol, *M*_w_ = 13,240/8840 g/mol, *Ð*^e^ = 3.74/2.58, β-O-4 (A) = 56.31/59.52%, phenylcoumarans (B) = 7.13/5.68%, resinol (C) = 13.56/4.16%, cinnamyl alcohol end groups (I) = 0/0%, S/G/H (unit) = 69.00/30.62/0.38% (60.97/35.79/3.24%)	RT-800 °C^a^	800 °C^d^—char: PiL (39.33%), PoL (30.19%), BaL (20.71%)	400–600 °C^b^: PiL (guaiacol), PoL (phenol), BaL (4-vinylphenol)	[110]
Sugarcane bagasse (BG)	Organosolv method (ethanol)	N/A	BG-Lignin	G/S/H-type lignin with G/S/H (unit ratio) = 0.82/0.02/0.16; moisture = 3.78%, volatiles = 67.34%, fixed carbon = 28.80%, ash = 0.09%, phenolic OH/aliphatic OH = 0.79	RT-400, 500, 600, 700 °C^c^	800 °C^d^—char (29%)	400 °C^c^ (2,6-dimethoxy-phenol); 500 °C–600 °C^c^ (2,6-dimethoxy-4-(2-propenyl)-phenol); 700 °C^c^ (4-ethyl-phenol)	[216]
Willow biomass	Organosolv method (ethyl acetate-EAC/tetrahydrofuran-THF/γ-butyrolactone-GBL)	N/A	L-EAC (83.8%), THF (92.3%), GBL (72.6%)	L-EAC/L-THF/L-GBL: G-type lignin with C = 65.00/66.25/66.88%, H = 6.26/6.03/6.32%, N = 0.22/0.30/0.21%, O = 28.09/27.00/26.61%, ash = 0.44/0.12/0%, moisture = 4.5/4.0/4.5%	RT-500 °C^a^	500 °C^a^—gas/oil/char: L-EAC (22.15/34.53/43.32%), L-THF (20.97/35.21/43.83%), L-GBL (21.70/33.55/44.75%)	500 °C^b^: L-EAC, L-THF (guaiacol, 4-methylguaiacol), L-GBL (4-methylguaiacol)	[220]

^a^A fixed bed

^b^GC/MS and/or GC/FID (gas chromatograph/flame ionization detection)

^c^Py-GC/MS

^d^TG-DTG

^e^Dispersity

^f^U-type tube pyrolysis device

^g^In situ FTIR spectroscopy

^h^Tube furnace

^i^TG–MS (thermogravimetry–mass spectrometry)

**Table 2:** Structures and isolations of DES lignin, and corresponding pyrolytic behaviors

Raw material	DES synthesis	Isolation	Lignin	Lignin properties	Pyrolysis temperature	Pyrolytic property		Refs.
						Yield	Major compositions in oils	
*Populus deltoides*	DESs: ChCl coupled with urea and imidazole with molar ratio of 1:2 and 3:7, followed by heating at 60 °C until a homogeneous liquid formed	Lignin regenerated and purified from pretreated Populus by DESs (115/150 °C, 15 h) via ethanol/water (1:9, v:v)	Lyophilized lignin labeled as ChU-115 (26.4%), ChI-115 (27.0%), ChI-150 (12.1%)	ChU-115/ChI-115/ChI-150: G/S-type lignin with *M*_w_ = 2562–1544 g/mol, *Ð*^e^ ≤ 1.60, ASL = 7.23/8.18/3.5%, AIL = 19.2/18.9/8.5%, total carbohydrates = 5.26/3.76/1.65%, aliphatic OH = 3.61/3.65/3.60 mmol/g, total phenolic OH = 1.18/0.94/1.28 mmol/g, COOH = 1.18/0.94/1.28 mmol/g, β-O-4 = 50.3/48.3/46.9%, β–β = 10.9/13.7/6.4%, β-5 = 5.5/2.5/2.8%, S/G = 1.38/1.42/2.27	RT-650 °C^a^	600 °C^b^—char (ChI-115 > ChI-150 > ChU-115)	650 °C^a^: ChI-115, ChI-150, ChU-115 (phenol)	[240]
Oil palm empty fruit bunch (EFB)	DESs: choline chloride/lactic acid (CC-LA) with molar ratio of 1:1 and 1:15, and choline chloride/formic acid (CC-FA) with molar ratio of 1:2, heated at 80 °C until homogeneous phase	Lignin isolated and purified from pretreated EFB using DESs (120 °C, 8 h) via ethanol/water (1:2, v:v)	DES-extracted lignin (DEEL) by LA/CC-LA (1:1)/CC-LA (1:15)/CC-FA	LA/CC-LA (1:1)/CC-LA (1:15)/CC-FA-Lignin: G/S/H-type lignin with lignin content = 75.15/78.89/80.64.85.84%, glucan = 1.42/1.94/0.00/1.39%, xylan = 5.94/0.62/1.02/0.00%, C = 52.87/52.50/53.89/47.86%, H = 5.78/5.39/5.17/5.44%, N = 0.00/1.36/0.77/2.48%, O = 41.98/40.00/38.80/42.83%, S = 0.49/0.75/1.37/1.40%, G (unit) = 13.97/29.79/49.91/35.65%, S (unit) = 83.85/64.78/50.09/62.01%, H (unit) = 2.18/5.43/0/2.34%, methoxy = 14.16/10.32/29.04/25.95%	RT-650 °C^a^	900 °C^b^—char (CC-FA > CC-LA 1:15 > CC-LA 1:1 > LA)	650 °C^a^: LA, CC-LA (1:1), CC-LA (1:15), CC-FA (phenol)	[241]
Commercial softwood lignin (SL)	DESs: ChCl/EG (CE), ZnCl_2_/EG (ZE), ChCl/Aa (CA) with the same molar ratio of 1:2 at 60 °C	Lignin treated by DESs at 120 °C for 2 h and collected by acid precipitation	CEL, ZEL, CAL	CAL/CEL/ZEL: G/H-type lignin with C = 63.05/63.11/62.95%, H = 5.85/6.15/6.02%, N = 0.13/0.13/0.13%, O = 30.04/29.48/29.93%, S = 0.49/0.75/1.37/1.40, HHV = 25.76/26.22/25.94 MJ/kg, H/C_eff_ = 0.38/0.45/0.42, *M*_w_ = 2650/2574/2529 g/mol, *M*_n_ = 1233/1238/1204 g/mol, *Ð*^e^ = 2.15/2.08/2.10, β-O-4 = 2.9/1.7/0.3, β–β = 2.3/2.2/2.4, β-5 = 4.0/3.9/4.1, G-type OH = 2.23/2.16/2.05 mmol/g, C-type OH = 0.29/0.26/0.40 mmol/g, H-type OH = 0.06/0.11/0.05 mmol/g, condensed G-type OH = 2.15/2.12/2.14 mmol/g, aliphatic OH = 0.85/1.07/1.10 mmol/g, COOH = 0.65/0.54/0.46 mmol/g, methoxy = 4.02/3.76/3.45 mmol/g	RT-550 °C^a^	900 °C^a^-gas/oil/char: CEL, ZEL, CAL (15/35/50%)	550 °C^a^: CEL (phenol, *p*-cresol, guaiacol, creosol), ZEL (creosol, catechol, guaiacol), CAL (guaiacol, creosol, catechol, toluene)	[229]
Eucalyptus tenuifolia	DESs: choline chloride/formic acid (FDES) and choline chloride/lactic acid (ADES) with the same molar ratio of 1:10, heated at 60–80 °C until homogeneous phase	Raw material extracted by toluene/ethanol (2:1, v:v) for 6–8 h, followed by DESs treatment at 110 °C for 2, 4, 6 h, and lignin isolated and purified by acetone/water (7:3, v:v)	A2, A4, A6, F2, F4, F6	A2/A4/A6/F2/F4/F6: G/S-type lignin with glucan = 5.69/3.34/12.17/0/1.12/0.23%, xylan = 9.25/8.86/13.29/8.11/4.96/4.98%, galactan = 1.34/1.33/1.86/3.64/1.64/1.50%, C = 58.85/58.24/58.79/60.24/59.86/60.84%, H = 5.72/5.75/5.60/5.70/5.94/5.75%, N = 0.28/0.35/0.21/0.95/1.66/1.02%, O = 34.28/35.33/35.18/29.86/29.50/30.59%, methoxy = 23.15/22.86/21.57/23.25/23.50/22.50%	RT-800 °C^c^	800 °C^b^—char: F4 > A4	200–600 °C^c^: products rich in aromatic structures, of which the functional groups are temperature-induced to form light gases, H_2_O, CO_2_ CO, and CH_4_	[236]

^a^Py-GC/MS

^b^TG-DTG

^c^TG-FTIR

**Table 3:** Structures and isolations of biological lignin, and corresponding pyrolytic behaviors

Raw material	Enzyme	Isolation	Lignin	Lignin properties	Pyrolysis temperature	Pyrolytic property		Refs.
						Yield	Major compositions in oils	
Softwood (Fir), hardwood (Eucalyptus), non-wood (moso-bamboo)	Industrial cellulase (CMCase)	Enzymatic treatment (hydrolysis) of biomass using CMCase at 40 °C for 48 h, followed by lignin collection via centrifugation and purification via mild acidolysis in dioxane/acidified water (0.01 mol/L HCl, 85:15, v:v) at 87 °C	Softwood/hardwood/non-wood EMAL lignin (> 50%)	Softwood EMAL lignin: G/H-type lignin with G (unit) = 91.2%, H (unit) = 2.8%, β-O-4 = 56.1%, α-O-4 and β-5 = 19.0%, β–β = 5.3%, β-1 = 2.9% Hardwood/non-wood EMAL lignin: G/S/H-type lignin with G (unit) = 24.7/36.1%, S (unit) = 54.6/42.9%, H (unit) 1.5/21.0%, β-O-4 = 36.3/38.7%, α-O-4 and β-5 = 4.4/5.2%, β–β = 9.6/6.0%, β-1 = 2.0/0%	RT-300, 400, 500, 600 °C^a^	800 °C^b^—char: ~ 30%	300 °C^b^: softwood EMAL lignin (coniferyl alcohol, sinapyl alcohol); 400 °C^b^: hardwood EMAL lignin (coniferyl alcohol, sinapyl alcohol); 400 °C^b^: non-wood EMAL lignin (4-vinylphenol, 4-vinylguaiacol, 4-vinylsringol)	[126]
Ginkgo, poplar	White-rot fungus *Physisporinus vitreus*	Lignin isolated from biomass using Björkman method and modified by white-rot fungus at 30 °C and 150 rpm for 48 h	Ginkgo lignin-laccase, poplar lignin-laccase	Ginkgo lignin-laccase, poplar lignin-laccase: G/H-type lignin with acid soluble lignin (ASL) = 0.32/0.29%, Klason lignin = 97.26/97.05%, carbohydrates = 1.18/1.32%, ash = 0.38/0.41%, C = 59.98/58.89%, H = 6.01/5.57%, N = 0.43/0.15%, O = 33.32/35.21%, S = 0.26/0.18%, O/C (molar ratio) = 0.42/0.45, H/C (molar ratio) = 1.20/1.13, methoxy = 11.17/15.68%	RT-400, 600, 800 °C^c^	600 °C (exp.)^c^—gas/oil/char: Ginkgo lignin-laccase (13.8/14.5/43.3%), poplar lignin-laccase (15.5/11.4/40.7%)	600 °C (exp.)^c^: Ginkgo lignin-laccase (2-methoxy-phenol, vanillin2-methoxy-4-methyl-phenol), poplar lignin-laccase (2,6-dimethoxy-phenol, 2-methoxy-phenol, isoeugenol)	[249]
Four technical lignin [sugarcane bagasse steam explosion (SE), sugarcane bagasse soda-anthraquinone (SAQ), sodium lignosulfonate from eucalyptus (NaE) and sodium lignosulfonate from a mixture of eucalyptus and pine wood (NaPE)]	Lignin peroxidase (LiP), quinone reductase (OR)	Lignin treated by LiP and OR in sodium tartrate buffer (pH 3.0) at 25 °C for 24 h	SE-Lignin, SAQ-Lignin, NaE-Lignin, NaPE-Lignin	SE-Lignin, SAQ-Lignin, NaE-Lignin, NaPE-Lignin: G/S/H-type lignin with *M*_w_ = 10,126/6087/10,833/9023 g/mol, *M*_n_ = 800/1112/3165/4351 g/mol, *Ð*^e^ = 12.7/5.5/3.4/2.1	RT-550 °C^d^	550 °C^d^—gas/oil/char: SE-E-Lignin (8.8/47.4/43.8%), SAQ-E-Lignin (22.0/40.6/37.3%), NaE-E-Lignin (21.2/27.5/51.3%), NaPE-E-Lignin (36.4/19.8/43.8%)	550 °C^e^: SE-Lignin, SAQ-Lignin, NaE-Lignin (phenol), NaPE-Lignin (guaiacol)	[255, 256]
Bamboo	White-rot fungus *Echinodontium taxodii*	Enzymatic hydrolysis performed in sodium acetate buffer (pH = 4.8) at 48 °C for 3 days, and lignin isolated by centrifugation	Lignin from enzymatic treated EHRL (86.4%)	G/S-type lignin with G/S (unit ratio) = 0.49, and with the decreasing content of β-O-4 structure	RT-600 °C^a^	1100 K^f^-char: 31.64, 32.18, 33.04% at 40, 20, 10 K/min	600 °C^a^: 2,3-dihydrobenzofuran, syringol, vanillic acid	[257]

^a^Py-GC/MS

^b^GC-FID

^c^A fixed bed reactor

^d^A stainless steel tubular reactor

^e^GC/MS

^f^TG/DTG

**Table 4:** Structures and isolations/treatments of ionic liquid lignin, and corresponding pyrolytic behaviors

Raw material	Ionic liquid	Isolation/treatments	Lignin	Lignin properties	Pyrolysis temperature	Pyrolytic property		Refs.
						Yield	Major compositions in oils	
Empty fruit bunches (EFB), palm mesocarp fiber (PMF) and palm kernel shells (PKS)	Pyridinium formate [PyFor]	Biomass pretreated by [PyFor], and lignin regenerated via dissolving in acetone/water followed by acetone evaporation	EFB lignin, PMF lignin, PKS lignin	EFB lignin, PMF lignin, PKS lignin: with C = 42.02/42.95/45.59%, H = 6.48/5.70/6.21%, N = 5.74/6.56/7.02%, O = 44.59/44.63/40.90%, S = 0.18/0.16/0.28%, acid insoluble lignin = 17.82/32.23/45.54%, HHV = 27.05/27.40/28.20 MJ/kg	50–550 °C^a^	800 °C (10 °C/min)^b^—char (exp.): EFB lignin (3.87%), PMF lignin (5.79%), PKS lignin (9.25%)	550 °C^a^: EFB lignin (cyclotetradecane, naphthalene, 4-t-butylcyclohexanone), PMF lignin (naphthalene, 4-t-butylcyclohexanone, phenol), PKS lignin (phenol, 2-methoxy-phenol, 2,4-dimethylphenol)	[271, 274]
Sugarcane straw (SCS)	1-Ethyl-3-methylimidazolium acetate [Emin][OAc]	SCS pretreated by [Emin][OAc] at 90 °C for 5 h, and lignin recovered via dissolving in acetone/water followed by acetone evaporation	Recovered lignin	N/A	50–700 °C^c^	700 °C—char^d^: 44%	350 °C^c^: phenols, methanol, formaldehyde	[125]
Industrial lignin	1-Butyl-3-methylimidazolium chloride (BMIC)	Lignin pretreated in a microwave apparatus using BMIC at 50, 100, 150 °C for 30 min and precipitated using water	BMIC-lignin_50_, BMIC-lignin_100_, BMIC-lignin_150_	N/A	RT-300, 400, 500, 600, 700, 800°C^e^	300–400 °C^e^-liquid (exp.): BMIC-lignin_50_ (~ 0.150 g/g_lignin_), BMIC-lignin_100_ (~ 0.125 g/g_lignin_), BMIC-lignin_150_ (~ 0.125 g/g_lignin_)	300–500 °C^f^: BMIC-lignin_50_, BMIC-lignin_100_, BMIC-lignin_150_ (phenolics, arenes, alkanes)	[280]
Industrial lignin	1-Sulfonic acid butyl-3-methylimidazolium trifluoromethanesulfonate [B(SO_3_H)min]-OTf	Lignin pretreated in a microwave apparatus using [B(SO_3_H)min]-OTf at 50, 100, 150, 200 °C for 1 h under N_2_, and regenerated by washing with water	Lignin-50, lignin-100, lignin-150, lignin-200	Industrial lignin: ash = 3.77%, volatiles = 56.73%, C = 66.22%, H = 6.30%, O = 25.58%, N = 1.09%, S = 0.81%	RT-300, 400, 500, 600, 800°C^e^	300–400 °C^e^—tar (exp.): Lignin-50 (0.1889 g/g_lignin_) lignin-100 (0.1231 g/g_lignin_) lignin-150 (0.1338 g/g_lignin_) lignin-200 (0.0751 g/g_lignin_)	300–500 °C^f^: Lignin-50, lignin-100, lignin-150, lignin-200 (phenol, guaiacol, 2-ethylphenol, 4-methoxyguaiacol)	[279]
Industrial lignin	1-Butyl-3-methylimidazolium dihydrogen phosphate [Bmin]H_2_PO_4_	Lignin pretreated in a microwave reactor using [Bmin]H_2_PO_4_ at 50, 100, 150 °C for 30 min, and washed out by water	Lignin-50, lignin-100, lignin-150	Industrial lignin: moisture = 2.6%, ash = 2.5%, volatiles = 62.4%, C = 63.9%, H = 5.6%, O = 28.6%, N = 1.1%, S = 0.8%	RT-300, 400, 500, 800 °C^e^	300–400 °C^e^-liquid (exp.): Lignin-50 (~ 0.15 g/g_lignin_) lignin-100 (~ 0.12 g/g_lignin_) lignin-150 (~ 0.12 g/g_lignin_)	300–500 °C^f^: lignin-50, lignin-100, lignin-150 (phenolics, arenes, alkanes)	[270]

^a^Py-GC/MS

^b^TG-DTG

^c^TG-FTIR

^d^TG-DTG

^e^A vertical fixed-bed quartz reactor

^f^GC/MS

#### Kraft lignin

Kraft lignin (a kind of technical lignin) is generated from Kraft pulping process in the pulp and paper industries from black liquor, among which sulfide solutions (i.e., sulfuric acid) is commonly used [177]. In the delignification process, hydrogen sulfide would react with lignin. Although these sulfur-containing components would be decomposed at the later stage of cooking process, the formed elemental sulfur can combine with hydrogen sulfide to produce polysulfide, which still leads to 2–3% sulfur content in the Kraft lignin [178]. For example, organic sulfur-containing species in Kraft lignin are presented in forms of free sulfur, sulfides, sulfites, sulfones, sulfates, disulfides, polysulfides, thiols, etc. [179, 180]. Han et al. investigated the evolution of sulfur-containing compounds during Kraft lignin pyrolysis [181]. During pyrolysis process, the formed sulfur-containing compounds in pyrolytic products can be presented in forms of H_2_S, SO_2_, CH_3_SH, CH_3_SCH_3_, and CH_3_SSCH_3_. On the other hand, the role of inorganic sulfur compounds in the pyrolysis of Kraft lignin was reported by Dondi et al. that the presence of sulfates as oxidizers can increase the amount of CO_2_ formed at high pyrolytic temperatures (> 200 °C), higher than the theoretical value of CO_2_ production from carboxyl groups in lignin [182]. However, the remaining sulfur content in Kraft lignin is disadvantageous for its application on pyrolysis due to the acidity of generated oils and air pollution during pyrolysis due to the release of foul-smelling sulfur compounds [152, 183]. Furthermore, sulfur contaminations would also inhibit the depolymerization of Kraft lignin [184]. Therefore, before being used in pyrolysis, sulfur removing would be necessary to improve the oil production. For example, many methods, such as adsorption, sodium hydroxide treatment, the oxidation of thiols and sulfides to sulfoxides and sulfones followed by organosolv extraction, direct sulfur extraction by organic solvents, recovering organic sulfur compounds by P(III)-based systems as well as elemental sulfur recovery with heavy metals and sodium sulfite, and neutralization of acidic and basic sulfur-containing compounds to form salts, have been cited for reducing sulfur content [180].

The produced Kraft lignin mainly consists of guaiacyl- and syringyl-type units, and also contains considerable amounts of phenolic hydroxyl due to the extensive cleavage of β-O-4 linkage during pulping process [185, 186]. Therefore, massive studies have used Kraft lignin as pyrolytic subject to produce abundant phenolic products in recent years. Zhang et al. investigated the pyrolytic behaviors of synthetic Kraft lignin by TG–FTIR (thermogravimetric–Fourier transform infrared spectrometry) and Py-GC/MS techniques, and the resulted volatiles include H_2_O, CO_2_, CO, alkyls, carbonyls, alkenes, and aromatics (where guaiacol is the main component) [187]. Fan et al. studied the pyrolysis process of a commercial Kraft lignin, and the maximum oil yield can be obtained at 550–600 °C. The further increase of pyrolytic temperature would cause a significant increase in gas production, while have no obvious effect on char generation. Among phenolic products, S-type phenols occupy the main proportion (i.e., ~ 70%) from 400 to 700 °C. Besides, the proposed radical-mediated reaction processes during pyrolysis was proposed including lignin homolysis at 400–450/450–600 °C (to form active lignin radicals, such as phenolics), and radicals recombination at 600–700 °C (to generate some low-molecular-weight gases and char/coke) [188]. Thermal treatment was performed to treat Kraft lignin before pyrolysis via Demuner et al. to investigate the pyrolytic behaviors of thermally treated lignin. Results show that the thermal treatment promotes demethylation and demethoxylation [189]. On the other hand, various fractionations (e.g., fractionations via organosolv method [135], ultrafiltration [154], and gradient acid precipitation [190]) were used for the treatment of Kraft lignin. For example, Chen et al. fractionated Kraft lignin into different lignin fractions (F1, F2, and F3) and further investigated their pyrolysis characteristics [135]. Results show that lignin fractions with different molecular weight distributions, linkages type, functional groups content, and S/G ratio can be obtained, where lignin with more methoxy contains the stronger cracking of β aryl ether bonds [191]. Thermal stability of lignin shows that the smaller molecular weight, the faster maximum degradation rate, and the less char residue generated, which indicate that lower molecular weight leads to worse thermal stability with lower activation energy. Lower temperatures are disadvantageous for monomers production while good for oils generation, and the opposite trend can be obtained at higher temperatures. Ultrafiltration fractionation also influences the distribution of molecular weight of Kraft lignin, where the high-molecular-weight lignin fraction favors the generation of G-type compounds, and the low-molecular-weight one prefers S-type compounds. Temperature rising would cause the cleavage of methoxy of G- and S-type compounds to produce H-type ones [154]. Similarly, the lignin fractionation via gradient acid precipitation also affects molecular weight, which has an obvious effect on relative content of formed products from lignin pyrolysis [190].

#### Alkali lignin

Alkali lignin is a byproduct obtained from paper industry, which is produced by alkaline pulping procedure for separating lignin from the alkali lignin waste containing cellulose and lignin using NaOH and Na_2_S, which contains a large distribution of molecular weight [192, 193]. During cooking process, NaOH is commonly used as the chemical reagent to separate and degrade lignin fraction, wherein the cleavage of phenolic α-O-4, and non-phenolic/phenolic β-O-4 linkages, and the combination of Na^+^ and phenolic hydroxyl/carboxyl via chemical bonding to form phenolic-Na^+^ and carboxyl-Na^+^ groups happen simultaneously [194, 195], leading to obvious changes of lignin structure and significant influences on lignin pyrolysis via reducing activation energy [196].

For figuring out the influence of Na^+^ species on lignin pyrolysis, Dalluge et al. investigated the effect of Na treatment on lignin char and volatiles formations during fast pyrolysis, suggesting that the Na treated lignin exhibits the increasing yields of char (due to the catalytic effect derived from Na species [197]), the enhanced gasification of lignin [198] and the highest selectivity to aromatic volatiles from lignin pyrolysis compared to other alkali metals (e.g., Li, K, Cs) [199]. According to the above studies, Na species bonded with organic phase (e.g., –ONa^+^ and –COONa^+^) is a key factor for enhancing the conversion of alkali lignin. Guo et al. investigated the effect of Na groups on the production of lignin pyrolysis products [198]. The effect of different Na chemical forms (including organic/inorganic bonding Na groups, Or-/Inor-Na) on the condensable product composition is obtained that both two chemical forms of Na have the significant catalytic effect on phenolic hydroxyls removing to enhance the yield of benzenes (e.g., methoxybenzene, 1,2-dimethoxy-benzene, and 1,2,3-trimethoxy-benzene), and also have a positive effect on cracking alkyl substituents. The corresponding mechanism can be proposed that the β-O-4 linkage in lignin can be cleaved easily at low temperatures to generate alkyl guaiacols, of which the alkyl groups would be further eliminated by Or-Na, and Inor-Na is beneficial for removing phenolic hydroxyl to generate benzenes. Therefore, the quality of lignin oils obtained from pyrolysis process can be improved significantly via effective oxygen elimination [200, 201]. For the generation of light gases, both two Na chemical forms reduce the cracking of ether bond and methoxy to form CO and CH_4_, respectively. For condensed phase, char conversion with temperature indicates that both two Na chemical forms benefit gasification reaction due to their significant catalytic effects [202]. For Or-Na loaded char, char conversion rate is mainly influenced by the relative distribution of Na on char surface. At lower temperatures, the strong bonding strength of O–Na linkages inhibits the migration of Na element to the porous surface of char, leading to low catalytic activity. At higher temperatures, O–Na bonding strength becomes weaker, which is beneficial for Na adsorbing on char surface for enhancing catalytic activity [203]. For Inor-Na (i.e., NaCl) loaded char, the retention of Cl^−^ greatly affects the catalytic activity of Na^+^ on char conversion. Besides, with increasing temperature, Na^+^ tends to combine with CO_2_ (which is formed from lignin pyrolysis) to generate Na_2_CO_3_, which significantly deteriorates the catalytic activity [203].

Expect for Na^+^ species, other parameters, like temperature, reaction time, and molecular weight, also have significant effects on alkali lignin pyrolysis. For example, the effect of temperature and residence time on thermal decomposition reactions during fast pyrolysis of a softwood alkali lignin was investigated by Supriyanto et al. and the results show that there is a general increase in the yield of organic volatiles (of which the majority is G-type phenols) with the increase of temperature from 400 to 600 °C, wherein the cleavage of the lignin polymeric structure to form linear carbonyl (acetaldehyde) and G-type aromatic compounds is enhanced by increasing temperature [39]. On the other hand, the promoted effect of residence time (from 0.5 to 5 s) on lignin pyrolysis is significant at a lower temperature (i.e., 500 °C), while the extension of residence time at a higher temperature (i.e., 600 °C) would lead to more secondary cracking reactions to inhibit the further increase of aromatic volatile compounds generation. Biswas et al. demonstrated that increasing temperature benefits oil production, and the higher temperature prefers to alkyl phenols and catechols via demethylation, demethoxylation, and deoxygenation [204]. Guo et al. investigated the effects of molecular weight on the pyrolysis products properties of alkali lignin, and the results show that the molecular weight of alkali lignin is not significantly influenced on the temperature and species of products evolution, while is positively affected on the yields of CH_4_, CO, phenols, aromatic products. The lower molecular weight benefits the cracking of methoxy of lignin to generate CH_4_, CO, CO_2_, phenol, and alkyl phenol, but the higher molecular weight favors the generation of guaiacol and alkyl guaiacol. Besides, the molecular weight of formed aromatic compounds is influenced by both lignin molecular weight and pyrolysis temperature [205].

#### Organosolv lignin

Due to the low requirement for strong acids or bases, sulfur absence, simple downstream separation, and solvent recycling, organosolv fractionation using ethanol (the most commonly used) [206–209], ethyl acetate [210], acetone [211], etc., can be used as an efficient and economical method to separate lignin from lignocellulosic biomass. In organosolv fractionation, cellulose fraction can be remained in solid phase, and removed via filtration. Lignin fraction can be separated via precipitation (e.g., by adding excessive water in the obtained previous liquid containing hemicellulose and lignin fractions) and collected by centrifugation/filtration, which contains better solubility (in many organic solvents) and higher purity with lower structural modification compared to those of lignin obtained Kraft and soda processes [212].

Yuan et al. obtained lignin from different lignocellulosic biomass materials, like pine (softwood), eucalyptus (hardwood), and corn stalk (herbaceous feedstock), and bamboo (herbaceous feedstock) via organosolv fractionation using 96% dioxane as organic solvent, and investigated the structural effect on phenolic production [158]. Herbaceous-derived lignin shows higher molecular weight and dispersity compared to other lignin obtained from softwood and hardwood, suggesting the generation of heterogeneous lignin structure [213]. Thermal stability is directly related to lignin structure (e.g., the content of methoxy [191]), which indicates that the higher dispersity, the lower yield of char residue, suggesting the worse thermal stability [214]. For deeply understanding the correlation between lignin structure and phenolic production, lignin pyrolysis was conducted at 500 °C, and the results show that lignin can be mainly decomposed into phenolic compounds, of which the distribution is significantly influenced by different lignin types. The majority of 4-vinylphenol (H-type phenol) can be obtained from the pyrolysis of herbaceous lignin (i.e., from corn stalk and bamboo) due to the dominant H-type structure and abundant β-O-4 linkage in herbaceous lignin after organosolv fractionation, and demethylation and demethoxylation reactions during pyrolysis (to convert G-/S-type phenolic hydroxyls into H-type one via reducing the content of methoxy) [110, 215]. Soongprasit et al. used sugarcane bagasse (BG) as raw material for lignin separation (i.e., BG-lignin) using organosolv method (95% ethanol) and investigated its phenolic production from fast pyrolysis using Py-GC/MS technique [216]. With the temperature rising, several interconversion reactions are all enhanced, such as (1) S-/G-type phenols converted to H-type ones via demethoxylation reaction of methoxy; (2) H-type phenols converted to aromatic hydrocarbons via dehydroxylation reaction [217, 218]; (3) monolignol compounds converted to hydrocarbons via dealkylation [127]. On the other hand, temperature rising also makes a significant influence on phenolic selectivity via enhancing the cleavage of methoxy and other functional aromatic substituent groups [219], leading to the generation of oxygenated-alkyl methoxy phenols (OR-Ph-OCH_3_), alkyl methoxy phenols (R-Ph-OCH_3_), methoxy phenols (Ph-OCH_3_), alkyl phenols (R-Ph), and phenols (Ph), where (1) the oxygen elimination from OR-Ph-OCH_3_ at C_4_ positon via deoxygenation would form R-Ph-OCH_3_ and Ph-OCH_3_; (2) the cleavage of methoxy from Ph-OCH_3_ via demethoxylation would generate Ph and/or R-Ph (especially at high temperatures due to the high BDE of methoxy); and (3) the gasification of Ph via secondary cracking would produce hydrocarbons or other oxygenated volatiles [163]. Wang et al. investigated the influence of solvent fractionation (including ethyl acetate-EAC, tetrahydrofuran-THF, and γ-butyrolactone-GBL fractionations) on lignin pyrolysis [220], finding that different structures of lignin separated using different organic solvents via organosolv fractionation (i.e., EAC lignin contains the most oxygen-containing functional groups, GBL-lignin has the highest content of large-molecule fragments, and THF-lignin possesses the highest *T*_max_ at 398 °C) result in the different pyrolytic behaviors. For example, during pyrolysis, more intense cleavage of C_α_–C_β_ linkage can be obtained in GBL-lignin, while the C_1_–C_α_ linkage of in EAC- and THF-lignin is more fragile to be broken. After pyrolysis, (1) EAC-lignin mainly yields more guaiacol and 4-methylguaiacol via the cleavage of C_1_–C_α_ and C_α_–C_β_ linkages, (2) THF-lignin produces more guaiacol, 4-ethylguaiacol, and 4-methylguaiacol via cleaving C_1_–C_α_, C_β_–C_γ,_ and C_α_–C_β_ linkages, and (3) GBL-lignin generated the dominant 4-methylguaiacol via breaking the C_α_–C_β_ linkage.

#### DES (deep eutectic solvents) lignin

Deep eutectic solvents (DES), composed of hydrogen bond donor and acceptor, is an eco-friendly, economical and non-toxic method used for lignin extraction [224–227]. DES can reduce lignin molecular weight via cleaving weak bonding linkages, like β-O-4, which is conductive to improve the subsequent lignin pyrolysis efficiency [135, 228]. Three different DESs [i.e., choline chloride/acetic acid (CA), choline chloride/ethylene glycol (CE), and zinc chloride/ethylene glycol (ZE)] were used by Li et al. to modify softwood Kraft lignin (SKL) for improving the pyrolytic products distribution [229]. TG-DTG analysis shows significant difference of lignin pyrolytic behaviors during the temperature of 250–600 °C, where the maximum degradation temperature (*T*_max_) of DES treated lignin increases, indicating the higher thermal stability caused by the removal of unstable chemical linkages, such as β-O-4. The decreasing content of lignin char after pyrolysis (especially for lignin treated by CE) indicates that DES treatment is beneficial for char inhibition. The volatile products distribution shows that G-type phenols are the major compounds due to the nature of Kraft lignin extracted from black liquor [230, 231]. Among all phenolic compounds, G- and H-type phenols are generated by lignin side-chain fragmentation and free radical polymerization, while C-type phenols are mainly derived from side-chain fracture and thermal demethylation of other types of monophenols [232, 233]. During pyrolysis, G-type phenols can be gradually transformed into H-type ones due to the enhanced cleavage of methyl aryl ether bond caused by DES treatment. On the other hand, a small amount of mono aromatic hydrocarbons (MAHs) can also be observed, formed via secondary reactions, such as dehydroxylation and demethoxylation at high temperatures [234]. In addition, compared to untreated lignin, DES-treated samples can produce more MAHs due to their improved hydrogen to carbon efficient ratio (i.e., H/C_eff_) [235]. Furthermore, more phenolic products containing carbon number between C6 and C9 can be produced from DES-treated lignin pyrolysis, suggesting the positive effect of DES treatment on the generation of monophenols.

Li et al. extracted lignin with ChCl-formic acid/lactic acid systems (FDES/AEDS) for the comparative investigation of lignin structure, thermal stability, and pyrolytic products distribution [236]. The results show that lignin molecular weight can be reduced through DES treatment via initial lignin depolymerization [237], such as β-O-4 dissociation. On the other hand, DES treatment can also dissociate lignin–carbohydrate complex (LCC) and lignin substructures (e.g., aryl ether and carbon–carbon bonds) to purify lignin and make structural degradation. During pyrolysis, different stages can be observed, including drying stage (from 25 to 150 °C, including moisture removing) and pyrolysis stage (from 200 to 350 °C, mainly involving β-O-4 degradation; from 350 to 400 °C, mainly involving lignin side-chain oxidation; > 400 °C, mainly involving aromatic rings saturation and carbon–carbon bonds breaking; 400–600 °C, mainly involving the cleavage of methoxy group) [238], and lignin extracted from ADES contains more side chains and lower thermal stability compared to those of FDES extracting lignin. Volatiles releasing during pyrolysis shows that some low-molecular-weight gaseous products (e.g., CO, CO_2_, CH_4_, and H_2_O) are formed from the escape of functional groups outside the aromatic rings of lignin, where methoxy, methyl, and methylene are the main sources of CH_4_, CO_2_ is formed by decarboxylation and/or decarbonylation, CO is formed by the cleavage of ether bonds, and H_2_O mainly comes from dehydroxylation [239]. Furthermore, the yield of aromatic products of lignin increases with richer contents of non-condensable gaseous products after DES treatment, indicating that DES treatment is beneficial for thermal decomposition of lignin during pyrolysis.

#### Biological lignin

Biological treatment (an environmentally friendly method with low energy consumption) of lignin with assistance of fungus or bacterium has been recently reported as an effective pathway to facilitate lignin pyrolysis, including increasing thermal conversion rate and pyrolytic selectivity of lignin to specific products, decreasing pyrolytic temperature and activation energy [242–244]. For example, bio-treated bamboo lignin using white-rot fungi contained lower thermal stability, which can be decomposed into more G-type phenols through fast pyrolysis [245]. Lignin generated from bio-treatments (e.g., using cellular ligninolytic enzymes like laccase and peroxidases) can obtain certain structural modifications, of which the mechanism is based on the lignin structure oxidation and degradation (e.g., lignin side chains oxidation, demethoxylation, demethylation, and some linkages cleavage) [246–248]. For deeply understanding the effect of biological treatment on lignin pyrolysis, Wang et al. used a white-rot fungi in the bio-treatment of softwood ginkgo and hardwood poplar, where ligninolytic enzymes modified lignin structure and enhanced pyrolytic efficiency [249]. For example, oil production can be significantly improved after lignin enzymatic modification at all reaction temperatures, which is due to the decreasing content of C–C bonds in treated lignin samples [250], leading to the enhanced depolymerization of lignin structure, especially at higher temperatures. Besides, ether bonds (which can be cleaved at lower temperatures) are dominant in lignin interunit linkages, which are much easier to be decomposed compared to C–C bonds [251, 252], resulting in the significant increase of bio-oil from lignin (e.g., at 400 °C, Ginkgo lignin treated by Laccase yielding 52.2% oils, higher than 49.8% oils obtained from untreated Ginkgo lignin; at 400 °C, Poplar lignin treated by Laccase producing 51.9% oils, higher than 46.5% oils obtained from untreated Poplar lignin [249]). Besides, bio-treatment can also reduce the content of methoxy via enzymatic oxidation [253] and demethylation [254], thus decreasing char yield at high temperatures (e.g., at 800 °C, Poplar lignin treated by Laccase generating 21.2% char, lower than 23.2% char obtained from untreated Poplar lignin [249]). The selectivity of specific products of lignin can be improved significantly after bio-treatment, where G-/S-type phenols obtained from treated Poplar lignin can be effectively transformed into phenol at 800 °C due to the further temperature-induced cracking, including demethylation, demethoxylation, decarbonylation, and dealkylation in side chains [168].

#### Ionic liquid lignin

The strong electrostatic interactions between ions endow ionic liquid (ILs) with negligible vapor pressure and low flammability, which is beneficial for their applications under high-vacuum conditions without contamination problems. Also, their nature and properties can be adjusted by changing the chemical structures of both cations and anions according to specific applications [258, 259]. On the other hand, ILs can be recovered from the pretreated mixture by adding anti-solvents (e.g., ethanol or H_2_O, which would cause the generation of impurities in recovered ILs) [260], reverse micelle method (which can extract almost pure [Mmim] and [DMP] from pretreated mixture), or membrane distillation (separating ILs and water) [261].

Some ILs have been used as solvents for lignocellulosic biomass pretreatment, such as 1-butyl-3-methylimidazolium chloride ([Bmin][Cl]), 1-hexyl-3-methylimidazolium chloride [Hmin][Cl], 1-octyl-3-methylimidazolium chloride ([Omin][Cl]) [262], 1-ethyl-3-methylimidazolium acetate [Emin][OAc] [263, 264], and 1-butyl-3-methylimidazolium acetate [Bmin][OAc] [265]. Due to the high solubility of lignin (of which the mechanism is proposed to be realized by breaking H-bonds in lignin and combining the aromatic nucleus and aliphatic chain of lignin [266]), low energy consumption, environmentally friendly, solvent recycling, and low cost for synthesis [267, 268], many ILs have been used for lignin extraction from biomass, such as [Bmin][Cl], [Bmin][OAc], 1-ethyl-3-methylimidazolium lysinate [Emin][Lys], triethylammonium hydrogen sulfate [TEA][HSO_4_], and *N*,*N*-dimethylethanolammonium succinate ([DMEAS]) [269]. On the other hand, IL-treated lignin sample can exhibit the improved yields of pyrolytic oils and phenolic compounds during lignin pyrolysis due to the cleavage of hydrogen, ester, and ether bonds in lignin through IL treatment [270]. Rashid et al. used a protic ionic liquid pyridinium formate [PyFor] to extract lignin from various palm biomass, such as empty fruit bunches (EFB), palm mesocarp fiber (PMF), and palm kernel shell (PKS) [271], and three kinetic models (i.e., Flynn–Wall–Ozawa (FWO), Kissinger–Akahira–Sunose (KAS), and Starink) were used to investigate the activation energy of [PyFor] extracted lignin. Thermal stability of different lignin samples depends on their functional groups, intermolecular bonds, extraction methods, and condensation degree [272]. For example, due to the presence of increased branching/condensed structures and higher lignin content with higher molecular weight, higher thermal stability can be obtained [273–275]. Furthermore, heating rate is another important factor for lignin pyrolysis, indicating that the increasing heating rate would increase the reaction temperature and reduce the reaction period by accelerating pyrolysis [265, 276, 277]. Except for the nature of IL-extracted lignin, the secondary intra-particle reactions are also important factors in exothermic behaviors and thermal runway, thus having a significant effect on the kinetic mechanism and the magnitude of activation energy during pyrolysis [274, 278]. Halder et al. confirmed that the IL treatment would lead to the cleavage of β-O-4 bond in lignin and then cause the improved generation of phenolic compounds from lignin pyrolysis [125]. Lei et al. also found that the tar obtained from the pyrolysis of 1-sulfonic acid butyl-3-methylimidazolium trifluoromethanesulfonate [B(SO_3_H)min][OTf]-treated lignin (of which the content of aryl ether increases and that of fatty ether decreases) is mainly phenols and phenol derivatives, accounting for > 90% of all compounds, wherein the improved yields can be obtained for phenol, cresol, guaiacol, 2-ethylphenol, 4-ethyl-2-methoxy-phenol, and 4-methoxyguaiacol in light tar (of which the yield increases by 1.5 times compared to that of untreated lignin) [279].

## Catalytic pyrolysis using zeolites

### Zeolite-driven lignin catalytic pyrolysis with high MAHs selectivity

Table 5 shows the recent progress of some effective catalysts used in lignin catalytic pyrolysis for producing aromatic hydrocarbons (AHs), mainly including zeolites, carbon-based, and some metal-based catalysts. During catalytic pyrolysis, the main reaction pathway is divided into two parts, including in situ and ex situ catalytic pyrolysis. The feature of in situ catalytic pyrolysis is that the catalyst is mixed with lignin and then put into reactor together for pyrolysis, while ex situ one refers to the process of direct pyrolysis of lignin to form pyrolytic volatiles and then contact with the catalyst to obtain secondary catalytic pyrolysis [72].

**Table 5:** Recent progresses of catalysts used in lignin catalytic pyrolysis, and their selectivity

Lignin	Catalyst	Catalytic pyrolysis	Reaction conditions	Products yield	Important findings	Product selectivity	Ref.
Kraft lignin	Aluminosilicate	In situ	450, 550, and 650 °C, 12,500–40,625 °C/s, 6 s, He	N/A	HZSM5-30 showed the most promising anti-coking performance and the highest selectivity to desired products, HZSM5-500 showed good diffusion and high reaction rate, and the anti-coking performance of HY/MCM41 was weaker than HZSM5	Hydrocarbons selectivity: HZSM5-30 > HY > HZSM5-300/MCM41-40/MCM41-Si	[311]
Beech wood lignin	Micro/meso porous ZSM-5	In/Ex situ	400, 500, and 600 °C, 20 min, N_2_	Organic phase: 15–35 wt%; char: ~ 40 wt%; gas: < 20 wt%	Both two catalysts exhibited excellent dealkoxylation/aromatization reactivity to yield more aromatics. Meso-ZSM-5 induces higher dealkoxylation reactivity, leading to higher selectivity to BTX aromatics without the increase of PAHs	MAHs selectivity: meso-ZSM-5 > ZSM-5	[312]
Cellulolytic enzyme lignin	Micro-meso ZSM-5	In-situ	873 K, 30 s	Char: ~ 30 wt% > coke: ~ 15 wt% > aromatics: ~ 8 wt% > phenolics: ~ 3 wt% > catechols: ~ 2 wt%	Mesoporous structure was beneficial for the diffusion of heavy phenols and modulation of pyrolysis products	AHs selectivity: C6 > C8 > C10+ ≈ C7 > C9+ > C14+	[281]
Rice straw lignin	Modified ZSM-5	In situ	450, 500, 550, and 600 °C	N/A	These ZSM-5 catalysts showed shape selectivity and acidity, beneficial for pyrolytic products distribution, and demethoxylation and dehydroxylation of oxygenates	Hydrocarbons selectivity: (without catalyst) oxygenates > phenols > PAHs > MAHs; (with ZSM-5) PAHs > MAHs ≈ oxygenates ≈ phenols; (with alkali ZSM-5) PAHs > MAHs > phenols > oxygenates; (with Ni-ZSM-5) MAHs ≈ PAHs > oxygenates > phenols. MAHs: naphthalenes with 40–60%	[47]
Commercial lignin	Modified HZSM-5	Ex situ	500 °C (I) and 450–600 °C (II), 20 °C/min, N_2_	Organic liquid: 17.5–22.7 wt%; solid: 42.7–43.2 wt%; gas: 19.2–19.8 wt%	HZSM-5 treated by organic alkali exhibited a coordinated micro/meso-proportion with proper size and acidity, of which the acidity would be further enhanced after cobalt incorporation, exhibiting better MAHs selectivity	Alkali treated HZSM-5 preferred phenols, while Co-alkali-HZSM-5 exhibited much higher selectivity to MAHs (38%) than phenols (23%)	[313]
Commercial lignin	HZSM-5/biochar	Ex situ	500 °C, 10 min, N_2_	Oil: 35 wt%; total AHs: 50 mg/g (maximum)	LC (lignin carbon) incorporating lignin depolymerization produced more phenols, which can be further transformed into aromatic hydrocarbons through HZSM-5	The production of AHs from lignin/LC/HZSM-5 was almost two times higher than lignin/HZSM-5 (from 30 to 50 mg/g)	[297]
Alkali lignin	ZSM-5/biochar	In situ	500 °C, 10 min, N_2_	Oil: ~ 20 wt%; char: ~ 40 wt%; gas: < 20 wt%; aromatics: > 30 mg/g	The addition of biochar enhanced bond breaking of lignin to yield more oils, and ZSM-5 acted as a selective aromatization to obtain higher content of aromatics	Total aromatics (12.32%) > methoxyphenols (5.17%) > alkylphenols (4.4%) > phenols (2.42%) > acid (0.85%)	[314]
Corn cob lignin	HZSM-5@Al-SBA-15	In situ	550 °C, 10 °C/min, N_2_	Gas: ~ 35 wt%, water: < 10 wt%; organic liquid: < 15 wt%	Mixed zeolite with tailored properties of acidity and porosity regulated the composition of AHs by pre-cracking and enhanced diffusion	AHs selectivity: MAHs (~ 40%) > PAHs (~ 25%) > phenols (~ 13%) > aliphatics (~ 8%) > methoxyphenols (~ 0%)	[293]
Commercial lignin	Pine-Mo_2_C	In situ	300, 400, and 500 °C, WHSV = 1 h^−1^, H_2_(N_2_)	Total AHs: 12.36 wt%; light gases: 29.68 wt% (maximum)	Pine carbon supported Mo_2_C catalyst showed good selective deoxygenation targeting C-O cracking to produce more monocyclic aromatic hydrocarbons	Deoxygenation rate: pine-Mo_2_C (100%); MAHs selectivity: toluene (98%) > others (benzene and xylenes, < 2%)	[298]
Enzymatic hydrolysis lignin	Nb_2_O_5_	In situ	500–650 °C, 0.5 min	Phenols: ~ 4 wt%; MAHs: ~ 6 wt%	Nb_2_O_5_ exhibited excellent deoxygenation ability to convert lignin into AHs, especially for MAHs	AHs selectivity: C6+C7+C8+C9+C9+ (MAHs, up to 90%) > C10+C11+C12+C12+ (PAHs, up to 8%)	[303]
Kraft lignin	Biochar, activated carbon	In situ	550 °C, 5 min, N_2_	Oil: 13.15 wt%; char: 58.76 wt%; gas: 39.74 wt% (maximum)	The catalytic effect of biochar was derived from surface sodium and alkali metals. The addition of AC resulted in the high-phenol-concentration oils production	Products selectivity: phenols > PAHs > oxygenates	[296]
Bagasse lignin	Ca_0.5_Pr_0.5_FeO_3_	In situ	20 °C/min, 2 h, N_2_	Oil: ~ 25 wt%; char: > 50 wt%; gas: > 40 wt% (maximum)	Guaiacols, syringols, and phenols were the main component in pyrolytic oils, and the content of light aliphatic hydrocarbons increased after catalysts addition	Products selectivity: Guaiacols > syringols > phenyl ethers > phenolics > phenyl ketones	[306]
Bagasse lignin	La_0.8_M_0.2_FeO_3_ (M = La, Ca, Sr, Ba)	In situ	600 °C, 10 °C/min, 2 h, N_2_	Oil: ~ 25.73 wt%; char: > 40.65 wt%; gas: > 42.89 wt% (maximum)	Perovskites improved the generation of aliphatic hydrocarbons via inhibiting decarboxylation and decarbonylation, and increased aryl oxygen-containing compound yield	LaFeO_3_, La_0.8_Ca_0.2_FeO_3_, La_0.8_Sr_0.2_FeO_3_, and La_0.8_Ba_0.2_FeO_3_ produced the maximum selectivity of phenolics (24.59%), syringols (25.78%), guaiacols (23.79%), syringols (22.47%), respectively	[307]

Among all catalysts, zeolites have been widely used in lignin catalytic pyrolysis due to their availability, low cost, thermal stability, acidity, porosity, and excellent catalytic selectivity and charring inhibition capacity [44, 281–284]. Huang et al. [48] used five types of parent zeolites [HZSM-5 (25), HZSM-5 (85), Al-MCM-41, HY, and USY] in lignin pyrolysis, among which HZSM-5 (25) is the most effective one to produce AHs (BTX), showing the highest selectivity of 56.56%. Kumar et al. found that Y-zeolite is the most effective one for demethoxylation and dehydroxylation of both small and bulky oxygenates in lignin catalytic pyrolysis as compared to mordenite and ZSM-5 [285]. Some modifications further enhance the catalytic activity and selectivity to AHs, such as alkali treatment and metal incorporation. After alkali treatment (i.e., NaOH), the pore properties and acidic sites density of zeolites are all changed, with the enhanced performance on cracking bulky oxygenates (such as phenols) to produce AHs with sharper selectivity [47, 286, 287]. After metal incorporation (Fe, Co, Ni, Cu, Zn, Ga) to form bi-functional zeolite catalyst, deoxygenation and aromatization reactions are enhanced to sharply increase the selectivity to AHs (or MAHs), due to the increased mesoporous surface area, average size of parent zeolites, and enhanced lignin mass-transfer efficiency. The modification of zeolites conducted by Jin et al. obtained the highest MAHs content of 29.3% over Co-HZSM-5, which is also attributed to its better inhibition of carbon deposition [288]. In addition, Ni-ZSM-5 obtained the maximum selectivity of hydrocarbons at 72% [47] and Fe-ZSM-5 obtained the maximum selectivity to C_2_H_4_ at 80% and naphthalene at ~ 50% [289]. ZrO_2_/ZSM-5 realized the significant reduction of oxygen content from 30 to 10 wt% (with a higher share of aromatics and lower heavy components) [290], Zn-HZSM-5 improved total aromatics from 31.1 to 46.1 mg/g [291], and double-layer metal supported core–shell (ZSM-5@MCM-41, Z@M) catalysts (Me_1_Z@Me_2_M) showed synergistic effect on the promotion of MAHs selectivity via phenols, immoderate aromatization, and polymerization inhibitions [292]. Mixed zeolite, like HZSM-5@Al-SBA-15, was confirmed with both pre-cracking and enhanced diffusion (Al-SBA-15), and shape AHs selectivity (HZSM-5), which can improve MAHs production while inhibit PAHs generation [284, 293].

Carbon-based materials, such as activated carbon (AC), has also been confirmed as effective catalysts in upgrading pyrolysis of lignin, due to their well-developed pore structures and abundant acidic surface groups. Furthermore, AC has been investigated as an effective catalyst for the selective generation of phenols (the heavy components in bio-oils) [294, 295]. Yang et al. also confirmed that AC (produced from steam activation of lignin char) has the great potential to be used as upgrading catalyst for lignin catalytic pyrolysis. The catalytic effect of AC derived from surface sodium and alkali metals, and the enhanced diffusion efficiency for reactive intermediates attributed to its inside mesopores were believed to be the important determinant of high activity and selectivity of AC to produce guaiacol-rich oils. Another important factor is its efficient inhibition to tar production and char agglomeration [296]. Therefore, many studies focused on the combination of carbon-based materials and zeolites, on one hand, to increase intermediates yield via cleaving C–O bonds on carbon-based materials, on the other hand, to generate more AHs via sharp selectivity of zeolites. For example, HZSM-5/lignin char catalyst prepared by Zhang et al. both showed high depolyermization of lignin (to generate more small-molecular-weight intermediates) and created the increased yield of total aromatics (from 29.5 to 50.1 mg/g). Besides, the addition of char can also alleviate the deactivation of HZSM-5 caused by coke deposition [297].

### Other catalysts-driven lignin catalytic pyrolysis

Other types of catalyst were also used in lignin pyrolysis (shown in Table 5), such as Mo_2_C, Nb_2_O_5_, and perovskites. Due to the similar properties to noble metal catalysts, transition metal carbides, which have good catalytic performance (such as C–O cleavage and deoxygenation abilities for Mo_2_C under H_2_ condition), were used in lignin catalytic pyrolysis. In situ pyrolysis products of biomass can be used as carbon source and supporter for carburizing Mo precursor to produce biomass-Mo_2_C, which would contain more activity to realize carbonization and metal reduction in one step [298]. On the other hand, lignin bonds breaking and deoxygenation can be achieved under normal pressure via biomass-Mo_2_C to obtain high yield of MAHs (13.26 wt%) with high selectivity to toluene (> 98%) and high deoxygenation ratio of > 95% [298]. Some metal oxides were also introduced in lignin catalytic pyrolysis, such as Fe_2_O_3_, CuO, ZnO, and CaO, which can also increase AHs yield and reduce heavy components in oils [299, 300]. Among these catalysts, niobium pentoxide (Nb_2_O_5_) has gained much attention in lignin catalytic pyrolysis both in batch- and fixed-bed reactions [301–303]. Lewis acid sites endow niobium pentoxide with high catalytic activity in hydrodeoxygenation of lignin or its derivatives [304], exhibiting high AHs yield at 11.2 wt% with extremely high selectivity of MAHs at 94% due to its efficient inhibition of PAHs and coke/char formation and effective deoxygenation ability [303]. Finally, perovskite-type oxides, due to their high activity, high stability, strong resistance to poisoning, low cost, and excellent properties of transporting oxygen ions and electrons, have played an important role in catalytic reactions [305]. They have also been selected as effective catalysts in lignin catalytic pyrolysis, such as Ca_0.5_Pr_0.5_FeO_3_ [306], La_0.8_M_0.2_FeO_3_ (M = La, Ca, Sr, Ba) [307], LaTi_x_Fe_1−x_O_3_ [308], and LaTi_0.2_Fe_0.8_O_3_ [309, 310]. The main component in bio-oils obtained after catalytic pyrolysis over perovskite-type oxides is aryl oxygen-containing compounds with high selectivity (from 60 to 80%, such as phenolics, guaiacols, syringols and phenylates) instead of MAHs, while the inhibition of decarboxylation and decarbonylation can reduce the generation of CO/CO_2_ and increase aliphatic hydrocarbons fracture on aromatic rings [307].

## Co-pyrolysis with other hydrogen-rich feedstock

According to the description above (“Catalytic pyrolysis using zeolites” section), zeolite catalysts are applied widely for improving the quality of lignin-based oils via deoxygenation to increase the production of AHs, although their yields are relatively low owing to the low H/C_effective_ ratio (or the low key hydrogen index) of mere lignin used [315]. On the other hand, the addition of catalysts would lead to the formation of coke which decreases the production of oils and affect the pyrolysis efficiency. The co-pyrolysis is a popular method to improve the oil quality via combination of lignin with other hydrogen-rich feedstock, such as the co-pyrolysis of lignin with plastics (a common hydrogen-rich substrate) [316, 317]. On the other hand, due to the massive agricultural, industrial and human activities, biomass and plastic wastes (e.g., polyethylene, polypropylene, polystyrene, polyvinylchloride, and polyethylene terephthalate [318–320]) have been remained with a huge production annually, which significantly requires us to take actions for their highly effective utilizations. Co-pyrolysis is a feasible and simple thermochemical technology for achieving the goal of high-efficiency utilization of these common wastes in our life due to the same heating system with pyrolysis technology without any significant adjustment [321]. Furthermore, blended substrates placed in the co-pyrolysis technique would produce more yields and wider distributions (i.e., MAHs, PAHs, and other long-chain hydrocarbons) of pyrolytic products as compared to those of single lignin used due to the synergistic effect (Table 6) [64, 322]. The quality of formed oils can be improved via co-feeding with plastics to increase the C/H content [323], and the heating value of condensable products obtained from co-pyrolysis would also be enhanced obviously [324]. Esso et al. summarized some important parameters, such as blending ratio, heating system (including heating rate and reaction temperature), and catalyst used on the synergy effect between biomass and co-feedings [316]. Lignin is hydrogen-deficient component in lignocellulosic biomass only with H/C_effective_ range of 0–0.3; hence, the hydrogen donors (such as low-density polyethylene, high-density polyethylene, phenol–formaldehyde resins, polyolefins, and spent bleaching clay) can be used for promoting hydroprocessing (such as hydrogenolysis and/or hydrogenation) of lignin oxygenates species to increase the yield of AHs [55, 325–330]. Typically, hydrogen radicals produced from the thermal degradation of hydrogen donors were beneficial for lignin oils and char upgrading via hydrocracking, hydrogenolysis, dehydration, oligomerization, and polymerization [64]. On the other hand, some studies also focused on the enhancement of oil production and char evolution via co-pyrolysis, such as lignin–collagen [331], lignin–cellulose [123, 124], lignin–waste oil [61], lignin–oil shale [332], lignin/cellulose/sawdust-coke bottle [333], and lignin–coal [334, 335], of which the detailed information is listed in Table 6. Through co-pyrolysis, not only lignin but also other kinds of wastes with low-efficient utilization can achieve their valorizations.

**Table 6:** Detailed information of lignin co-pyrolysis

Blending mixture	Catalyst	Reactor	Synergistic effect	Yield	Selectivity	Ref.
Lignin/low-density polyethylene (LDPE)	HZSM-5	A thermogravimetric analyzer	1. The synergistic effect decreased the starting temperature of pyrolysis and accelerated the decomposition rate 2. The composition of pyrolysis products contained more alkane and aromatic hydrocarbons	800 °C—char: lignin > lignin + LDPE > lignin + LDPE + HZSM-5 > LDPE	Lignin + LDPE + HZSM-5: aromatics, alkane^a^	[325]
Lignin/plastics (PS)	Red clay (RC)	A micro reactor	The synergistic effect increased the yield of guaiacol	900 °C^b^—char: lignin (14.9%), lignin/PS (6.04%), lignin/PS/RC (33.54%); 500 °C^c^—lignin-/PS-/lignin + PS-/lignin + PS + RC-derived compounds: 0.81/6.03/4.67/14.67%	Lignin (guaiacol, 60.41%), lignin/RC (guaiacol, 67.37%), lignin/PS (styrene-C8, 70.22%), lignin/PS/RC (styrene-C8, 86.86%)	[317]
Lignin/phenol–formaldehyde resins (PF)	HZSM-5	A vertical pyrolysis reactor	The synergistic effect improved the yield of aromatic amines	650 °C^c^—total compounds/aromatic amines: PF (24.5/14.2%), lignin (7.2/3.7%), lignin/PF = 1/5 (22.0/13.3%)	650 °C^c^: lignin (simple phenols, 75.62%, catechols, 14.49%, aromatic hydrocarbon, 9.89%) lignin/PF (simple phenols, 84.16%, dimers, 6.30%, aromatic hydrocarbons, 4.23%), PF (simple phenols, 83.2%, aromatic hydrocarbons, 8.6%, dimers, 6.80%)	[326]
Lignin/spent bleaching clay (BC)	N/A	A fixed-bed reactor system	1. The synergistic effect promoted the production of P-type phenols and aromatic hydrocarbons, and the inhibition of oxygenates species 2. The addition of spent bleaching clay reduced the activation energy and improve the oil quality	550 °C—gas/oil/char: BC (~ 9/25/66%), lignin (~ 34/24/42%), lignin/BC = 1/1 (~ 19/25/57%)	550 °C^c^: BC (aliphatic hydrocarbons), lignin (phenols), lignin/BC = 1/1 (H-type phenols)	[329]
Lignin/polyolefins	N/A	A fixed-bed reactor	1. Liquid yield was improved by adding polyolefins due to the formation of lower poly-hydrocarbons derived from polyolefins 2. The yield of gasoline and kerosene was climbed, revealing that lignin pyrolytic products enhanced the cracking of polyethylene (PE) intermediates 3. The rearrangement reactions of polypropylene (PP) intermediates enhanced liquid production via inhibiting the formation of solid	650 °C—gas/liquid/Wax/char: lignin/PE (~ 10/40/25/20%), lignin/PP (~ 10/50/15/20%)	Lignin + PE: hydrocarbons like > C26 and C19-C25, ad diesel (C14–C18)^c^; Lignin + PP: hydrocarbons like > C26 and C11–C13, and gasoline (C5–C10)^c^	[327]
Lignin/waste cooking oil (WCO)	HZSM-5	A Py-GCMS system	The addition of waste cooking oil in lignin with appropriate catalyst-to-feedstock ratio contributed to high selectivity to aromatics via enhancing alkylation and demethoxylation	823 K^c^—feedstock conversion rate: lignin (~ 65%, phenolics, MAHs), WCO (~ 100%, MAHs, PAHs), lignin/WCO = 1/1 (~ 85%, MAHs, PAHs)	823 K^c^: lignin (phenolics, MAHs, ~ 65%), lignin/WCO = 1/1 (MAHs, ~ 75%), WCO (MAHs, ~ 60%)	[61]
Lignin/collagen	N/A	An applied test system, Inc. series 3210 vertical tube furnace	1. Collagen addition benefited the formation and evolution of char with abundant oxygenated poly-aromatic structures 2. Collagen addition improved the hydrogen aromaticity index via reducing aromatic substitution and/or facilitating the removal of functional groups 3. Collagen acted as a binder for lignin to form stronger fused framework	600 °C—char: lignin (45.1%), lignin/collagen (45.4–48.4%), collagen (24.9%); 1000 °C—char: lignin (41.8%), lignin/collagen (42.2–43.6%), collagen (22.6%)	600 °C^d^—C:O/C:H/C:N (aromatic ratio of char): lignin (3.21/0.90/0), lignin/collagen (3.17/0.84/32.26), collagen (2.32/0.54/3.18)	[331]
Cellulose/lignin/sawdust	N/A	A self-made fixed-bed reactor	1. Interactions between the volatiles generated from cellulose and sawdust promoted oil production 2. Repolymerization happened between light species from cellulose and heavy species from lignin to generate more condensable liquids 3. Interaction between heavy species from lignin and sawdust created more carbonaceous char	500 °C—gas/oil/char: cellulose (28.1/55.6/16.3%), sawdust/cellulose (11.5/71.0/17.5%), sawdust (25.2/50.0/24.8%), lignin (8.5/39.6/51.9%), sawdust/lignin (17.5/41.5/41%)	500 °C^c^: lignin (phenols), cellulose (sugars, aldehydes, ketones), sawdust (sugars), sawdust/lignin (phenols, aldehydes, ketones), cellulose/sawdust (aldehydes, ketones), cellulose/lignin (sugars)	[123]

^a^TG-FTIR

^b^TG-DTG

^c^GC/MS, GC/FID

^d^Elemental analysis

## Condensing and separating processes of pyrolytic oils

The determination of oil compositions can be achieved by Py-GC/MS [222], and volatile evolutions can be detected by TG-FTIR [205] during the pyrolysis reaction. In addition, through dissolving oils in some specific solvents (e.g., isopropanol), GC/MS/FID, 2D-GC/FID, or NMR techniques can be used to obtain their chemical compositions/structures [116, 204]. Gooty et al. proposed a condensing system consisting of two cyclonic condensers (i.e., condensers 1 and 3), an electrostatic precipitator-cum-condenser (i.e., C-ESP), and a cotton wool filter for pyrolytic oils collection [336]. The generated vapor/gas flows through condenser 1, C-ESP, condenser 3, and the cotton wood filter in series, wherein the liquid yield can be obtained by measuring the weight of cyclonic condensers and C-ESP before and after each run, and oil deposit can be recovered with a specially designed scraper [337]. However, the complexity of oil composition makes the subsequent separation difficult. So far, solvent extraction is an efficient method for products separation. For example, Deng et al. performed the extraction of phenols from bio-oil aqueous fraction using hydrophobic ionic liquids (i.e., [Bmin][NTf_2_]), where the extraction efficiencies of acetic acid, phenol, guaiacol, and 2-methylguaiacol in aqueous fraction were 2.71, 95.41, 92.04, and 97.98% [338]. Yao et al. extracted and recovered phenolic compounds from aqueous solution by thermo-separating magnetic ionic liquid aqueous two-phase system (two polypropylene glycol, PPG600-based thermo-separating magnetic ionic liquid aqueous), where the extraction efficiencies of phenol, o-cresol, and m-cresol were higher than 95% [339]. A hydroxyl-functionalized ionic liquid (i.e., [C_2_OH-4-pic][NO_3_]) was used by Xu et al. to extract phenolic compounds from model coal tar, which obtained the extraction efficiency of 98.99, 98.39, 98.65, and 99.99% for m-cresol, o-cresol, p-cresol, and phenol, respectively [340]. A binary petroleum ether/dichloromethane solvent (PE/DCM) was used by Yang et al. to extract aromatic monomers from lignin oils, of which the extraction efficiency was 80% [341]. In addition, PAHs can be efficiently extracted by various techniques, such as supercritical and subcritical fluid extraction, microwave-assisted solvent extraction, plant oil-assisted extraction, and some microextraction techniques using less organic solvents [342].

## Summary and future perspective

In this review, we introduced pyrolysis technology for lignin conversion and further lignin oils upgrading via catalytic pyrolysis using zeolites and co-pyrolysis with other hydrogen-rich feedstock. Different lignin samples, such as Kraft lignin, alkali lignin, organosolv lignin, DES lignin, biological lignin, and ionic liquid lignin, and their corresponding pyrolytic behaviors were presented, which indicated the effect of lignin type, reaction temperature, and time on lignin pyrolytic properties. Lignin pyrolysis can be mainly divided into three stages (initial, primary and charring stages), where light gases (H_2_O, CO_2_, CO, CH_4_), phenolic compounds (G-/S-/H-/C-type phenols), and char with aromatic fused-ring structures can be obtained. In addition, zeolites and other catalysts (activated carbon, Mo_2_C, metal oxides, and perovskites) were used for lignin oils upgrading to generate AHs via deoxygenation. Finally, hydrogen-rich co-feedings were applied to form more AHs (MAHs, PAHs) via increasing H/C_eff_ ratio of blending substrates and generating hydrogen radicals from hydrogen donors during co-pyrolysis.

In spite of huge effort put on lignin pyrolysis, there still some challenges. Some suggestions are made below for the future perspective.

1. Lignin is a natural source of phenolic compounds, of which the production of G-/S-/H-/C-type phenolic compounds are majorly determined by the composition and content of corresponding phenolic units in lignin. For example, G-type phenolic compounds (e.g., guaiacol) can be significantly produced from G-type lignin. However, the complexity of lignin structure always leads to the complexity of formed phenolic products (mainly including G-/S-/H-type phenolic compounds). The efficient removal of methoxy from lignin can adjust and regularize lignin structure to improve the selectivity of generated phenolic compounds (which is beneficial for the formation of H-type phenolic compounds). On the other hand, the removal of methoxy can reduce the formation of light gases, which is beneficial for lignin conversion to liquid products.
2. Holocellulose can also be converted into phenolic compounds via the promotion of specific catalysts. Furthermore, electronegative products and radicals generated from lignin pyrolysis can be involved in the secondary reaction of polyhydroxy volatiles from holocellulose. Therefore, further investigations should be made on concerning the interactions between volatiles of lignin and holocellulose, which can be solved by isotope-labeled model compounds with typical functional groups together with the control of pyrolysis conditions.
3. For lignin catalytic pyrolysis, other capacities (except for deoxygenation) of catalysts used should be obtained, such as hydrogenation, alkylation, decarbonylation, and demethylation, to avoid the generation of phenolic compounds with distinct substituents. The development of promising catalysts used for lignin upgrading will never be an outdated researching topic for a long time.
4. Co-pyrolysis is an efficient method, and will be a main direction of lignin pyrolysis in the future. Hydrogen donors provided from other co-feedings are significantly beneficial for oil hydroprocessing to generate high yield of AHs. On the other hand, these co-feedings used in co-pyrolysis can be produced from agricultural, industrial, and human activities in large quantities annually. Therefore, lignin co-pyrolysis with other new co-feedings and their pyrolysis mechanisms should be investigated in the near future.
5. Hydrogen provided by lignin itself (self-hydrogen transfer) used for lignin hydrogenolysis has been a popular researching topic in recent years, which can also be applied in lignin pyrolysis with certainly controlling pyrolysis conditions, reactor designed, and/or catalysts used.

## Abbreviations

BDE: Bond dissociation energy
LDH: Layered double hydroxide
TGA: Thermogravimetric analysis
DTG: Derivative thermogravimetric analysis
Py-GC/MS: Pyrolysis-gas chromatograph/mass spectroscopy
AHs: Aromatic hydrocarbons
MAHs: Monocyclic aromatic hydrocarbons
PAHs: Polycyclic aromatic hydrocarbons
EHI: Effective hydrogen index
LGA: Levoglucosan
LGO: Levoglucone
HHV: High heating value
FTIR: Fourier transform infrared spectroscopy
GC/MS: Gas chromatography/mass spectrometry
TG-IR-GC/MS: Fourier transform infrared spectroscopy and gas chromatography–mass spectrometry
THF: Tetrahydrofuran
KL: Kraft lignin
TG–FTIR: Thermogravimetric–Fourier transform infrared spectrometry
AL: Alkali lignin
OL: Organosolv lignin
EAC: Ethyl acetate
GBL: γ-Butyrolactone
GC/FID: Gas chromatograph/flame ionization detection
TG–MS: Thermogravimetry–mass spectrometry
DES: Deep eutectic solvent
CA: Choline chloride/acetic acid
CE: Choline chloride/ethylene glycol
ZE: Zinc chloride/ethylene glycol
FDES: Choline chloride/formic acid
ADES: Chloride/lactic acid
LCC: Lignin–carbohydrate complex
ILs: Ionic liquids
FWO: Flynn–Wall–Ozawa
KAS: Kissinger–Akahira–Sunose
EFB: Empty fruit bunches
PMF: Palm mesocarp fiber
PKS: Palm kernel shell
SCS: Sugarcane straw
*Ð*: Dispersity
*T*_max_: The temperature with maximum degradation rate
G-/S-/H-: Guaiacyl-/syringyl-/*p*-hydroxyphenyl-
UV: Ultraviolet
2D-GC/FID: 2D-gas chromatograph/flame ionization detection
NMR: Nuclear magnetic resonance spectroscopy

## References

[1] Abu-Omar MM, Barta K, Beckham GT, Luterbacher JS, Ralph J, Rinaldi R (2021). Guidelines for performing lignin-first biorefining. Energy Environ Sci.

[2] Sethupathy S, Murillo Morales G, Gao L, Wang H, Yang B, Jiang J (2022). Lignin valorization: status, challenges and opportunities. Bioresour Technol.

[3] Radhika NL, Sachdeva S, Kumar M (2022). Lignin depolymerization and biotransformation to industrially important chemicals/biofuels. Fuel.

[4] Kim HU, Kim JW, Tran NT, Limarta SO, Ha J-M, Park Y-K (2022). Direct conversion of lignin to high-quality biofuels by carbon dioxide-assisted hydrolysis combined with transfer hydrogenolysis over supported ruthenium catalysts. Energy Convers Manag.

[5] Yang L, Zheng T, Huang C, Yao J (2022). Using deep eutectic solvent pretreatment for enhanced enzymatic saccharification and lignin utilization of masson pine. Renew Energy.

[6] Roberto Paz Cedeno F, Belon de Siqueira B, Gabriel Solorzano Chavez E, Ulises Miranda Roldán I, Moreira Ropelato L, Paul Martínez Galán J (2022). Recovery of cellulose and lignin from Eucalyptus by-product and assessment of cellulose enzymatic hydrolysis. Renew Energy.

[7] Yuan Y, Jiang B, Chen H, Wu W, Wu S, Jin Y (2021). Recent advances in understanding the effects of lignin structural characteristics on enzymatic hydrolysis. Biotechnol Biofuels.

[8] Mahdavi M, Mirmohammadi M, Baghdadi M, Mahpishanian S (2022). Visible light photocatalytic degradation and pretreatment of lignin using magnetic graphitic carbon nitride for enhancing methane production in anaerobic digestion. Fuel.

[9] Li C, Zhao X, Wang A, Huber GW, Zhang T (2015). Catalytic transformation of lignin for the production of chemicals and fuels. Chem Rev.

[10] Chakar FS, Ragauskas AJ (2004). Review of current and future softwood kraft lignin process chemistry. Ind Crop Prod.

[11] Lin F, Ma Y, Sun Y, Song Z, Men X, Wu Y (2022). Selective hydrodeoxygenation of lignin model compound to renewable fuel precursors using two-dimensional nanosheet Ni/HZ5-NS catalyst. Renew Energy.

[12] Umar Y, Velasco O, Abdelaziz OY, Aboelazayem O, Gadalla MA, Hulteberg CP (2022). A renewable lignin-derived bio-oil for boosting the oxidation stability of biodiesel. Renew Energy.

[13] vom Stein T, den Hartog T, Buendia J, Stoychev S, Mottweiler J, Bolm C (2015). Ruthenium-catalyzed C–C bond cleavage in lignin model substrates. Angew Chem Int Ed.

[14] Nichols JM, Bishop LM, Bergman RG, Ellman JA (2010). Catalytic C–O bond cleavage of 2-aryloxy-1-arylethanols and its application to the depolymerization of lignin-related polymers. J Am Chem Soc.

[15] Liu Y, Li C, Miao W, Tang W, Xue D, Li C (2019). Mild redox-neutral depolymerization of lignin with a binuclear Rh complex in water. ACS Catal.

[16] Liu YX, Li CZ, Miao W, Tang WJ, Xue D, Xiao JL (2020). Rhodium–terpyridine catalyzed redox-neutral depolymerization of lignin in water. Green Chem.

[17] Jiang L, Guo HW, Li CZ, Zhou P, Zhang ZH (2019). Selective cleavage of lignin and lignin model compounds without external hydrogen, catalyzed by heterogeneous nickel catalysts. Chem Sci.

[18] Wu K, Wang W, Guo H, Yang Y, Huang Y, Li W (2020). Engineering co nanoparticles supported on defect MoS_2–x_ for mild deoxygenation of lignin-derived phenols to arenes. ACS Energy Lett.

[19] Liu H, Li H, Lu J, Zeng S, Wang M, Luo N (2018). Photocatalytic cleavage of C–C bond in lignin models under visible light on mesoporous graphitic carbon nitride through π–π stacking interaction. ACS Catal.

[20] Liu H, Li H, Luo N, Wang F (2020). Visible-light-induced oxidative lignin C–C bond cleavage to aldehydes using vanadium catalysts. ACS Catal.

[21] Nguyen JD, Matsuura BS, Stephenson CRJ (2014). A photochemical strategy for lignin degradation at room temperature. J Am Chem Soc.

[22] Liu E, Segato F, Prade RA, Wilkins MR (2021). Exploring lignin depolymerization by a bi-enzyme system containing aryl alcohol oxidase and lignin peroxidase in aqueous biocompatible ionic liquids. Bioresour Technol.

[23] Li Y, Cai Z, Liao M, Long J, Zhao W, Chen Y (2017). Catalytic depolymerization of organosolv sugarcane bagasse lignin in cooperative ionic liquid pairs. Catal Today.

[24] Mehta MJ, Kulshrestha A, Sharma S, Kumar A (2021). Room temperature depolymerization of lignin using a protic and metal based ionic liquid system: an efficient method of catalytic conversion and value addition. Green Chem.

[25] Prado R, Brandt A, Erdocia X, Hallet J, Welton T, Labidi J (2016). Lignin oxidation and depolymerisation in ionic liquids. Green Chem.

[26] Dier TKF, Rauber D, Durneata D, Hempelmann R, Volmer DA (2017). Sustainable electrochemical depolymerization of lignin in reusable ionic liquids. Sci Rep.

[27] Liu X, Bouxin FP, Fan J, Budarin VL, Hu C, Clark JH (2020). Recent advances in the catalytic depolymerization of lignin towards phenolic chemicals: a review. ChemSusChem.

[28] Schutyser W, Renders T, Van den Bosch S, Koelewijn SF, Beckham GT, Sels BF (2018). Chemicals from lignin: an interplay of lignocellulose fractionation, depolymerisation, and upgrading. Chem Soc Rev.

[29] Questell-Santiago YM, Galkin MV, Barta K, Luterbacher JS (2020). Stabilization strategies in biomass depolymerization using chemical functionalization. Nat Rev Chem.

[30] Rinaldi R, Jastrzebski R, Clough MT, Ralph J, Kennema M, Bruijnincx PCA (2016). Paving the way for lignin valorisation: recent advances in bioengineering, biorefining and catalysis. Angew Chem Int Ed.

[31] Liao Y, Koelewijn S-F, Van den Bossche G, Van Aelst J, Van den Bosch S, Renders T (2020). A sustainable wood biorefinery for low-carbon footprint chemicals production. Science.

[32] Zhou C-H, Xia X, Lin C-X, Tong D-S, Beltramini J (2011). Catalytic conversion of lignocellulosic biomass to fine chemicals and fuels. Chem Soc Rev.

[33] Xu C, Arancon RAD, Labidi J, Luque R (2014). Lignin depolymerisation strategies: towards valuable chemicals and fuels. Chem Soc Rev.

[34] Shen X, Zhang C, Han B, Wang F (2022). Catalytic self-transfer hydrogenolysis of lignin with endogenous hydrogen: road to the carbon-neutral future. Chem Soc Rev.

[35] Yu J, Sun L, Berrueco C, Fidalgo B, Paterson N, Millan M (2018). Influence of temperature and particle size on structural characteristics of chars from Beechwood pyrolysis. J Anal Appl Pyrol.

[36] Asmadi M, Kawamoto H, Saka S (2011). Gas- and solid/liquid-phase reactions during pyrolysis of softwood and hardwood lignins. J Anal Appl Pyrol.

[37] Figueiredo MB, Hita I, Deuss PJ, Venderbosch R, Heeres H (2022). Pyrolytic lignin: a promising biorefinery feedstock for the production of fuels and valuable chemicals. Green Chem.

[38] Li C, Hayashi J-I, Sun Y, Zhang L, Zhang S, Wang S (2021). Impact of heating rates on the evolution of function groups of the biochar from lignin pyrolysis. J Anal Appl Pyrol.

[39] Supriyanto, Usino DO, Ylitervo P, Dou J, Sipponen MH, Richards T (2020). Identifying the primary reactions and products of fast pyrolysis of alkali lignin. J Anal Appl Pyrol.

[40] Zheng Q, Zhang D, Fu P, Wang A, Sun Y, Li Z (2022). Insight into the fast pyrolysis of lignin: unraveling the role of volatile evolving and char structural evolution. Chem Eng J.

[41] Tran QK, Le ML, Ly HV, Woo HC, Kim J, Kim S-S (2021). Fast pyrolysis of pitch pine biomass in a bubbling fluidized-bed reactor for bio-oil production. J Ind Eng Chem.

[42] Yu H, Wang S, Sun Y, Zhang W, Li R, Kang X (2022). Pyrolysis mechanism law of β-O-4 lignin dimer model compounds: a density functional theory study. Ind Crop Prod.

[43] Wang L, Yin J, Jiang J, Zhang Y, Song M, Zhang R (2022). Revealing G-lignin model compounds pyrolysis behavior: β-O-4 and 5–5′ dimer and trimer. Fuel.

[44] Paysepar H, Venkateswara Rao KT, Yuan Z, Shui H, Xu C (2020). Production of phenolic chemicals from hydrolysis lignin via catalytic fast pyrolysis. J Anal Appl Pyrol.

[45] Ryu HW, Lee HW, Jae J, Park Y-K (2019). Catalytic pyrolysis of lignin for the production of aromatic hydrocarbons: effect of magnesium oxide catalyst. Energy.

[46] Zheng A, Huang Z, Wei G, Zhao K, Jiang L, Zhao Z (2020). Controlling deoxygenation pathways in catalytic fast pyrolysis of biomass and its components by using metal-oxide nanocomposites. iScience.

[47] Nishu, Li Y, Liu R (2022). Catalytic pyrolysis of lignin over ZSM-5, alkali, and metal modified ZSM-5 at different temperatures to produce hydrocarbons. J Energy Inst.

[48] Huang M, Xu J, Ma Z, Yang Y, Zhou B, Wu C (2021). Bio-BTX production from the shape selective catalytic fast pyrolysis of lignin using different zeolite catalysts: relevance between the chemical structure and the yield of bio-BTX. Fuel Process Technol.

[49] Rahman MM, Liu R, Cai J (2018). Catalytic fast pyrolysis of biomass over zeolites for high quality bio-oil—a review. Fuel Process Technol.

[50] Lee HW, Kim TH, Park SH, Jeon J-K, Suh DJ, Park Y-K (2013). Catalytic fast pyrolysis of lignin over mesoporous Y zeolite using Py-GC/MS. J Nanosci Nanotechnol.

[51] Kim S-S, Lee HW, Ryoo R, Kim W, Park SH, Jeon J-K (2014). Conversion of kraft lignin over hierarchical MFI zeolite. J Nanosci Nanotechnol.

[52] Custodis VBF, Karakoulia SA, Triantafyllidis KS, van Bokhoven JA (2016). Catalytic fast pyrolysis of lignin over high-surface-area mesoporous aluminosilicates: effect of porosity and acidity. ChemSusChem.

[53] Serrano L, Cecilia JA, García-Sancho C, García A (2019). Lignin depolymerization to BTXs. Topics Curr Chem.

[54] Luo Z, Lu K, Yang Y, Li S, Li G (2019). Catalytic fast pyrolysis of lignin to produce aromatic hydrocarbons: optimal conditions and reaction mechanism. RSC Adv.

[55] Fan L, Chen P, Zhang Y, Liu S, Liu Y, Wang Y (2017). Fast microwave-assisted catalytic co-pyrolysis of lignin and low-density polyethylene with HZSM-5 and MgO for improved bio-oil yield and quality. Bioresour Technol.

[56] Xie Q, Addy M, Liu S, Zhang B, Cheng Y, Wan Y (2015). Fast microwave-assisted catalytic co-pyrolysis of microalgae and scum for bio-oil production. Fuel.

[57] Zhang X, Lei H, Zhu L, Qian M, Zhu X, Wu J (2016). Enhancement of jet fuel range alkanes from co-feeding of lignocellulosic biomass with plastics via tandem catalytic conversions. Appl Energy.

[58] Park Y-K, Jung J, Ryu S, Lee HW, Siddiqui MZ, Jae J (2019). Catalytic co-pyrolysis of yellow poplar wood and polyethylene terephthalate over two stage calcium oxide-ZSM-5. Appl Energy.

[59] Zhang H, Xiao R, Nie J, Jin B, Shao S, Xiao G (2015). Catalytic pyrolysis of black-liquor lignin by co-feeding with different plastics in a fluidized bed reactor. Bioresour Technol.

[60] Sophonrat N, Sandström L, Zaini IN, Yang W (2018). Stepwise pyrolysis of mixed plastics and paper for separation of oxygenated and hydrocarbon condensates. Appl Energy.

[61] Fan L, Ruan R, Li J, Ma L, Wang C, Zhou W (2020). Aromatics production from fast co-pyrolysis of lignin and waste cooking oil catalyzed by HZSM-5 zeolite. Appl Energy.

[62] Wang Y, Akbarzadeh A, Chong L, Du J, Tahir N, Awasthi MK (2022). Catalytic pyrolysis of lignocellulosic biomass for bio-oil production: a review. Chemosphere.

[63] Zhong S, Zhang B, Liu C, Shujaa Aldeen A, Mwenya S, Zhang H (2022). A minireview on catalytic fast co-pyrolysis of lignocellulosic biomass for bio-oil upgrading via enhancing monocyclic aromatics. J Anal Appl Pyrol.

[64] Zhang X, Lei H, Chen S, Wu J (2016). Catalytic co-pyrolysis of lignocellulosic biomass with polymers: a critical review. Green Chem.

[65] Kan T, Strezov V, Evans TJ (2016). Lignocellulosic biomass pyrolysis: a review of product properties and effects of pyrolysis parameters. Renew Sustain Energy Rev.

[66] Balagurumurthy B, Bhaskar T (2014). Hydropyrolysis of lignocellulosic biomass: state of the art review. Biomass Convers Biorefin.

[67] Patel M, Kumar A (2016). Production of renewable diesel through the hydroprocessing of lignocellulosic biomass-derived bio-oil: a review. Renew Sustain Energy Rev.

[68] Acharya B, Sule I, Dutta A (2012). A review on advances of torrefaction technologies for biomass processing. Biomass Convers Biorefin.

[69] Homagain K, Shahi C, Luckai N, Sharma M (2014). Biochar-based bioenergy and its environmental impact in Northwestern Ontario Canada: a review. J Forestry Res.

[70] Bridgwater AV (2012). Review of fast pyrolysis of biomass and product upgrading. Biomass Bioenergy.

[71] Mohan D, Pittman CU, Steele PH (2006). Pyrolysis of wood/biomass for bio-oil: a critical review. Energy Fuel.

[72] Qiu B, Tao X, Wang J, Liu Y, Li S, Chu H (2022). Research progress in the preparation of high-quality liquid fuels and chemicals by catalytic pyrolysis of biomass: a review. Energy Convers Manag.

[73] Chen Y, Fang Y, Yang H, Xin S, Zhang X, Wang X (2019). Effect of volatiles interaction during pyrolysis of cellulose, hemicellulose, and lignin at different temperatures. Fuel.

[74] Jiang L, Luo J, Xu F, Qian L, Wang Y, Li H (2022). High yield production of levoglucosan via catalytic pyrolysis of cellulose at low temperature. Fuel.

[75] Gu X, Ma X, Li L, Liu C, Cheng K, Li Z (2013). Pyrolysis of poplar wood sawdust by TG-FTIR and Py–GC/MS. J Anal Appl Pyrol.

[76] Lin Y-C, Cho J, Tompsett GA, Westmoreland PR, Huber GW (2009). Kinetics and mechanism of cellulose pyrolysis. J Phys Chem C.

[77] Patwardhan PR, Brown RC, Shanks BH (2011). Product distribution from the fast pyrolysis of hemicellulose. ChemSusChem.

[78] Leng E, Guo Y, Chen J, Liu S, Jiaqiang E, Xue Y (2022). A comprehensive review on lignin pyrolysis: mechanism, modeling and the effects of inherent metals in biomass. Fuel.

[79] Zhao C, Jiang E, Chen A (2017). Volatile production from pyrolysis of cellulose, hemicellulose and lignin. J Energy Inst.

[80] Zhang Y, Chen P, Liu S, Peng P, Min M, Cheng Y (2017). Effects of feedstock characteristics on microwave-assisted pyrolysis—a review. Bioresour Technol.

[81] Tsai W, Lee M, Chang Y (2007). Fast pyrolysis of rice husk: product yields and compositions. Bioresour Technol.

[82] Lazzari E, Schena T, Primaz CT, da Silva Maciel GP, Machado ME, Cardoso CAL (2016). Production and chromatographic characterization of bio-oil from the pyrolysis of mango seed waste. Ind Crop Prod.

[83] Isahak WNRW, Hisham MW, Yarmo MA, Hin T-YY (2012). A review on bio-oil production from biomass by using pyrolysis method. Renew Sustain Energy Rev.

[84] Jung S-H, Kang B-S, Kim J-S (2008). Production of bio-oil from rice straw and bamboo sawdust under various reaction conditions in a fast pyrolysis plant equipped with a fluidized bed and a char separation system. J Anal Appl Pyrol.

[85] Zheng J-l, Zhu X-f, Guo Q-x, Zhu Q-s (2006). Thermal conversion of rice husks and sawdust to liquid fuel. Waste Manag.

[86] Ji-lu Z (2007). Bio-oil from fast pyrolysis of rice husk: yields and related properties and improvement of the pyrolysis system. J Anal Appl Pyrol.

[87] Zheng J-L, Yi W-M, Wang N-N (2008). Bio-oil production from cotton stalk. Energy Convers Manag.

[88] Alvarez J, Lopez G, Amutio M, Bilbao J, Olazar M (2014). Bio-oil production from rice husk fast pyrolysis in a conical spouted bed reactor. Fuel.

[89] Abdullah N, Gerhauser H (2008). Bio-oil derived from empty fruit bunches. Fuel.

[90] Volli V, Singh RK (2012). Production of bio-oil from de-oiled cakes by thermal pyrolysis. Fuel.

[91] Encinar JM, González JF, González J (2000). Fixed-bed pyrolysis of *Cynara cardunculus* L. product yields and compositions. Fuel Process Technol.

[92] Encinar JM, Beltrán FJ, Ramiro A, González JF (1998). Pyrolysis/gasification of agricultural residues by carbon dioxide in the presence of different additives: influence of variables. Fuel Process Technol.

[93] Islam MR, Parveen M, Haniu H (2010). Properties of sugarcane waste-derived bio-oils obtained by fixed-bed fire-tube heating pyrolysis. Bioresour Technol.

[94] Pattiya A, Suttibak S (2012). Production of bio-oil via fast pyrolysis of agricultural residues from cassava plantations in a fluidised-bed reactor with a hot vapour filtration unit. J Anal Appl Pyrol.

[95] Kim SW, Koo BS, Ryu JW, Lee JS, Kim CJ, Lee DH (2013). Bio-oil from the pyrolysis of palm and Jatropha wastes in a fluidized bed. Fuel Process Technol.

[96] Apaydin-Varol E, Pütün E, Pütün AE (2007). Slow pyrolysis of pistachio shell. Fuel.

[97] Makibar J, Fernandez-Akarregi AR, Amutio M, Lopez G, Olazar M (2015). Performance of a conical spouted bed pilot plant for bio-oil production by poplar flash pyrolysis. Fuel Process Technol.

[98] Biswas B, Pandey N, Bisht Y, Singh R, Kumar J, Bhaskar T (2017). Pyrolysis of agricultural biomass residues: comparative study of corn cob, wheat straw, rice straw and rice husk. Bioresour Technol.

[99] Pattiya A (2011). Bio-oil production via fast pyrolysis of biomass residues from cassava plants in a fluidised-bed reactor. Bioresour Technol.

[100] Oasmaa A, Solantausta Y, Arpiainen V, Kuoppala E, Sipila K (2010). Fast pyrolysis bio-oils from wood and agricultural residues. Energy Fuel.

[101] Li J, Chen Y, Yang H, Zhu D, Chen X, Wang X (2017). Correlation of feedstock and bio-oil compound distribution. Energy Fuel.

[102] Huang X, Cao J-P, Shi P, Zhao X-Y, Feng X-B, Zhao Y-P (2014). Influences of pyrolysis conditions in the production and chemical composition of the bio-oils from fast pyrolysis of sewage sludge. J Anal Appl Pyrol.

[103] Ly HV, Kim S-S, Woo HC, Choi JH, Suh DJ, Kim J (2015). Fast pyrolysis of macroalga *Saccharina japonica* in a bubbling fluidized-bed reactor for bio-oil production. Energy.

[104] Pütün E, Ateş F, Pütün AE (2008). Catalytic pyrolysis of biomass in inert and steam atmospheres. Fuel.

[105] Ates F, Işıkdağ MA (2008). Evaluation of the role of the pyrolysis temperature in straw biomass samples and characterization of the oils by GC/MS. Energy Fuel.

[106] Ly HV, Kim S-S, Choi JH, Woo HC, Kim J (2016). Fast pyrolysis of *Saccharina japonica* alga in a fixed-bed reactor for bio-oil production. Energy Convers Manag.

[107] Wang J, Liu Z, Li J, Yan B, Tao J, Cheng Z (2022). In-situ hydrodeoxygenation of lignin via hydrothermal liquefaction with water splitting metals: comparison between autocatalytic and non-autocatalytic processes. Int J Hydrog Energy.

[108] Zhang L, Zhang S, Hu X, Gholizadeh M (2021). Progress in application of the pyrolytic lignin from pyrolysis of biomass. Chem Eng J.

[109] Du X, Wu S, Li T, Yin Y, Zhou J (2022). Ozone oxidation pretreatment of softwood kraft lignin: an effective and environmentally friendly approach to enhance fast pyrolysis product selectivity. Fuel Process Technol.

[110] Wang T-P, Li H, Yuan J-M, Li W-X, Li K, Huang Y-B (2021). Structures and pyrolytic characteristics of organosolv lignins from typical softwood, hardwood and herbaceous biomass. Ind Crop Prod.

[111] Demirbaş A (2002). Analysis of liquid products from biomass via flash pyrolysis. Energy Sources.

[112] Luo Z, Wang S, Guo X (2012). Selective pyrolysis of Organosolv lignin over zeolites with product analysis by TG-FTIR. J Anal Appl Pyrol.

[113] Hu C, Liu C, Liu Q, Zhang H, Wu S, Xiao R (2020). Effects of steam to enhance the production of light olefins from ex-situ catalytic fast pyrolysis of biomass. Fuel Process Technol.

[114] Chen W-H, Wang C-W, Ong HC, Show PL, Hsieh T-H (2019). Torrefaction, pyrolysis and two-stage thermodegradation of hemicellulose, cellulose and lignin. Fuel.

[115] Sharma A, Kaur P, Singh G, Arya SK (2021). Economical concerns of lignin in the energy sector. Clean Eng Technol.

[116] Ghalibaf M, Alén R, Hita I, Deuss PJ, Jan Heeres H, de Wild P (2022). Valorization potential of technical lignins from Norway spruce (*Picea abies*) via pyrolysis. J Anal Appl Pyrol.

[117] Mahmood N, Yuan Z, Schmidt J, Xu C (2016). Depolymerization of lignins and their applications for the preparation of polyols and rigid polyurethane foams: a review. Renew Sustain Energy Rev.

[118] Lee HV, Hamid SBA, Zain SK (2014). Conversion of lignocellulosic biomass to nanocellulose: structure and chemical process. Sci World J.

[119] Raveendran K, Ganesh A, Khilar KC (1996). Pyrolysis characteristics of biomass and biomass components. Fuel.

[120] Deng W, Xu K, Xiong Z, Chaiwat W, Wang X, Su S (2019). Evolution of aromatic structures during the low-temperature electrochemical upgrading of bio-oil. Energy Fuel.

[121] Xiong Z, Guo J, Chaiwat W, Deng W, Hu X, Han H (2020). Assessing the chemical composition of heavy components in bio-oils from the pyrolysis of cellulose, hemicellulose and lignin at slow and fast heating rates. Fuel Process Technol.

[122] Lu X, Zhu X, Guo H, Que H, Wang D, Liang D (2020). Investigation on the thermal degradation behavior of enzymatic hydrolysis lignin with or without steam explosion treatment characterized by TG-FTIR and Py-GC/MS. Biomass Convers Biorefin.

[123] Li C, Sun Y, Dong D, Gao G, Zhang S, Wang Y (2021). Co-pyrolysis of cellulose/lignin and sawdust: Influence of secondary condensation of the volatiles on characteristics of biochar. Energy.

[124] Chua YW, Wu H, Yu Y (2021). Effect of cellulose–lignin interactions on char structural changes during fast pyrolysis at 100–350 °C. Proc Combust Inst.

[125] Halder P, Kundu S, Patel S, Parthasarathy R, Pramanik B, Paz-Ferreiro J (2019). TGA-FTIR study on the slow pyrolysis of lignin and cellulose-rich fractions derived from imidazolium-based ionic liquid pre-treatment of sugarcane straw. Energy Convers Manag.

[126] Lei M, Wu S, Liang J, Liu C (2019). Comprehensive understanding the chemical structure evolution and crucial intermediate radical in situ observation in enzymatic hydrolysis/mild acidolysis lignin pyrolysis. J Anal Appl Pyrol.

[127] Liu C, Hu J, Zhang H, Xiao R (2016). Thermal conversion of lignin to phenols: relevance between chemical structure and pyrolysis behaviors. Fuel.

[128] Yang J, Wang X, Shen B, Hu Z, Xu L, Yang S (2020). Lignin from energy plant (*Arundo donax*): pyrolysis kinetics, mechanism and pathway evaluation. Renew Energy.

[129] Qiao Y, Wang B, Ji Y, Xu F, Zong P, Zhang J (2019). Thermal decomposition of castor oil, corn starch, soy protein, lignin, xylan, and cellulose during fast pyrolysis. Bioresour Technol.

[130] Wu Z, Zhu X, Guo H, Jiang Y, Gu X (2019). A kinetic study of lignin pyrolysis over base catalyst during steam exploded depolymerization. Catal Today.

[131] Yeo JY, Chin BLF, Tan JK, Loh YS (2019). Comparative studies on the pyrolysis of cellulose, hemicellulose, and lignin based on combined kinetics. J Energy Inst.

[132] Ding Y, Zhang W, Yu L, Lu K (2019). The accuracy and efficiency of GA and PSO optimization schemes on estimating reaction kinetic parameters of biomass pyrolysis. Energy.

[133] Hung C-M, Chen C-W, Huang C-P, Yang Y-Y, Dong C-D (2022). Suppression of polycyclic aromatic hydrocarbon formation during pyrolytic production of lignin-based biochar via nitrogen and boron co-doping. Bioresour Technol.

[134] Yu J, Wang D, Sun L (2021). The pyrolysis of lignin: Pathway and interaction studies. Fuel.

[135] Chen S, Cheng H, Wu S (2020). Pyrolysis characteristics and volatiles formation rule of organic solvent fractionized kraft lignin. Fuel.

[136] Lyu G, Wu Q, Li T, Jiang W, Ji X, Yang G (2019). Thermochemical properties of lignin extracted from willow by deep eutectic solvents (DES). Cellulose.

[137] Akazawa M, Kato Y, Kojima Y (2016). Application of two resinols as lignin dimer models to characterize reaction mechanisms during pyrolysis. J Anal Appl Pyrol.

[138] Hosoya T, Kawamoto H, Saka S (2009). Role of methoxyl group in char formation from lignin-related compounds. J Anal Appl Pyrol.

[139] Pu L, Wang X, Cao Q, Liu B, Liu H, Han Y (2019). Novel nonprecious metal loading multi-metal oxide catalysts for lignin depolymerization. Energy Fuel.

[140] Ma H, Li T, Wu S, Zhang X (2021). Demethylation of a methoxy group to inhibit repolymerization during alkaline lignin pyrolysis. Fuel.

[141] Li J, Bai X, Fang Y, Chen Y, Wang X, Chen H (2020). Comprehensive mechanism of initial stage for lignin pyrolysis. Combust Flame.

[142] Faravelli T, Frassoldati A, Migliavacca G, Ranzi E (2010). Detailed kinetic modeling of the thermal degradation of lignins. Biomass Bioenergy.

[143] Chua YW, Yu Y, Wu H (2019). Structural changes of chars produced from fast pyrolysis of lignin at 100–300 °C. Fuel.

[144] Shrestha B, le Brech Y, Ghislain T, Leclerc S, Carré V, Aubriet F (2017). A multitechnique characterization of lignin softening and pyrolysis. ACS Sustain Chem Eng.

[145] Wang Y, Li X, Mourant D, Gunawan R, Zhang S, Li C-Z (2012). Formation of aromatic structures during the pyrolysis of bio-oil. Energy Fuel.

[146] Thangalazhy-Gopakumar S, Adhikari S, Gupta RB, Fernando SD (2011). Influence of pyrolysis operating conditions on bio-oil components: a microscale study in a pyroprobe. Energy Fuel.

[147] Jiang G, Nowakowski DJ, Bridgwater AV (2010). Effect of the temperature on the composition of lignin pyrolysis products. Energy Fuel.

[148] Klemetsrud B, Eatherton D, Shonnard D (2017). Effects of lignin content and temperature on the properties of hybrid poplar bio-oil, char, and gas obtained by fast pyrolysis. Energy Fuel.

[149] Ma Z, Sun Q, Ye J, Yao Q, Zhao C (2016). Study on the thermal degradation behaviors and kinetics of alkali lignin for production of phenolic-rich bio-oil using TGA–FTIR and Py–GC/MS. J Anal Appl Pyrol.

[150] Mei Y, Zhang S, Wang H, Jing S, Hou T, Pang S (2020). Low-temperature deoxidization of lignin and its impact on liquid products from pyrolysis. Energy Fuel.

[151] Zhao S, Liu M, Zhao L, Zhu L (2018). Influence of interactions among three biomass components on the pyrolysis behavior. Ind Eng Chem Res.

[152] Lin X, Sui S, Tan S, Pittman CU, Sun J, Zhang Z (2015). Fast pyrolysis of four lignins from different isolation processes using Py-GC/MS. Energies.

[153] Jegers HE, Klein MT (1985). Primary and secondary lignin pyrolysis reaction pathways. Ind Eng Chem Process Des Dev.

[154] Shao L, Zhang X, Chen F, Xu F (2017). Fast pyrolysis of Kraft lignins fractionated by ultrafiltration. J Anal Appl Pyrol.

[155] Akhtar J, Amin NS (2012). A review on operating parameters for optimum liquid oil yield in biomass pyrolysis. Renew Sustain Energy Rev.

[156] Ansari KB, Arora JS, Chew JW, Dauenhauer PJ, Mushrif SH (2019). Fast pyrolysis of cellulose, hemicellulose, and lignin: effect of operating temperature on bio-oil yield and composition and insights into the intrinsic pyrolysis chemistry. Ind Eng Chem Res.

[157] Marathe PS, Westerhof RJM, Kersten SRA (2019). Fast pyrolysis of lignins with different molecular weight: experiments and modelling. Appl Energy.

[158] Yuan J-M, Li H, Xiao L-P, Wang T-P, Ren W-F, Lu Q (2022). Valorization of lignin into phenolic compounds via fast pyrolysis: impact of lignin structure. Fuel.

[159] Beste A, Buchanan AC (2009). Computational study of bond dissociation enthalpies for lignin model compounds. Substituent effects in phenethyl phenyl ethers. J Org Chem.

[160] Hu J, Shen D, Xiao R, Wu S, Zhang H (2013). Free-radical analysis on thermochemical transformation of lignin to phenolic compounds. Energy Fuel.

[161] Younker JM, Beste A, Buchanan Iii AC (2011). Computational study of bond dissociation enthalpies for substituted β-O-4 lignin model compounds. ChemPhysChem.

[162] McMillen DF, Malhotra R, Chang S-J, Ogier WC, Nigenda SE, Fleming RH (1987). Mechanisms of hydrogen transfer and bond scission of strongly bonded coal structures in donor-solvent systems. Fuel.

[163] Kawamoto H (2017). Lignin pyrolysis reactions. J Wood Sci.

[164] Feng Y, Wang JT, Liu L, Guo QX (2003). C–H and N–H bond dissociation energies of five-and six-membered ring aromatic compounds. J Phys Org Chem.

[165] Garcia-Perez M, Wang S, Shen J, Rhodes M, Lee WJ, Li C-Z (2008). Effects of temperature on the formation of lignin-derived oligomers during the fast pyrolysis of Mallee woody biomass. Energy Fuel.

[166] Ojha DK, Viju D, Vinu R (2017). Fast pyrolysis kinetics of alkali lignin: evaluation of apparent rate parameters and product time evolution. Bioresour Technol.

[167] Asatryan R, Bennadji H, Bozzelli JW, Ruckenstein E, Khachatryan L (2017). Molecular products and fundamentally based reaction pathways in the gas-phase pyrolysis of the lignin model compound p-coumaryl alcohol. J Phys Chem A.

[168] Dong C-Q, Zhang Z-F, Lu Q, Yang Y-P (2012). Characteristics and mechanism study of analytical fast pyrolysis of poplar wood. Energy Convers Manag.

[169] Patwardhan PR, Dalluge DL, Shanks BH, Brown RC (2011). Distinguishing primary and secondary reactions of cellulose pyrolysis. Bioresour Technol.

[170] Yang H, Dong Z, Liu B, Chen Y, Gong M, Li S (2021). A new insight of lignin pyrolysis mechanism based on functional group evolutions of solid char. Fuel.

[171] Jiang W, Wu S, Lucia LA, Chu J (2017). A comparison of the pyrolysis behavior of selected β-O-4 type lignin model compounds. J Anal Appl Pyrol.

[172] Jiang X-Y, Lu Q, Dong X-C, Hu B, Dong C-Q (2016). Theoretical study on the effect of the substituent groups on the homolysis of the ether bond in lignin trimer model compounds. J Fuel Chem Technol.

[173] Dong Z, Yang H, Chen P, Liu Z, Chen Y, Wang L (2019). Lignin characterization and catalytic pyrolysis for phenol-rich oil with TiO_2_-based catalysts. Energy Fuel.

[174] Xu J, Tang H, Su S, Liu J, Xu K, Qian K (2018). A study of the relationships between coal structures and combustion characteristics: the insights from micro-Raman spectroscopy based on 32 kinds of Chinese coals. Appl Energy.

[175] Li X, Hayashi J-I, Li C-Z (2006). Volatilisation and catalytic effects of alkali and alkaline earth metallic species during the pyrolysis and gasification of Victorian brown coal. Part VII. Raman spectroscopic study on the changes in char structure during the catalytic gasification in air. Fuel.

[176] Custodis VBF, Bährle C, Vogel F, van Bokhoven JA (2015). Phenols and aromatics from fast pyrolysis of variously prepared lignins from hard- and softwoods. J Anal Appl Pyrol.

[177] Vishtal AG, Kraslawski A (2011). Challenges in industrial applications of technical lignins. BioResources.

[178] Gierer J (1980). Chemical aspects of kraft pulping. Wood Sci Technol.

[179] Fenner RA, Lephardt JO (1981). Examination of the thermal decomposition of kraft pine lignin by Fourier transform infrared evolved gas analysis. J Agric Food Chem.

[180] Evdokimov AN, Kurzin AV, Fedorova OV, Lukanin PV, Kazakov VG, Trifonova AD (2018). Desulfurization of kraft lignin. Wood Sci Technol.

[181] Han T, Sophonrat N, Evangelopoulos P, Persson H, Yang W, Jönsson P (2018). Evolution of sulfur during fast pyrolysis of sulfonated Kraft lignin. J Anal Appl Pyrol.

[182] Dondi D, Zeffiro A, Speltini A, Tomasi C, Vadivel D, Buttafava A (2014). The role of inorganic sulfur compounds in the pyrolysis of Kraft lignin. J Anal Appl Pyrol.

[183] Beis SH, Mukkamala S, Hill N, Joseph J, Baker C, Jensen B (2010). Fast pyrolysis of lignins. BioResources.

[184] Daniel D, Khachatryan L, Astete C, Asatryan R, Marculescu C, Boldor D (2019). Sulfur contaminations inhibit depolymerization of Kraft lignin. Bioresour Technol Rep.

[185] Ibarra D, Chávez MI, Rencoret J, Del Río JC, Gutiérrez A, Romero J (2007). Lignin modification during Eucalyptus globulus Kraft pulping followed by totally chlorine-free bleaching: a two-dimensional nuclear magnetic resonance, Fourier transform infrared, and pyrolysis-gas chromatography/mass spectrometry study. J Agric Food Chem.

[186] Wen J-L, Sun S-L, Yuan T-Q, Sun R-C (2015). Structural elucidation of whole lignin from Eucalyptus based on preswelling and enzymatic hydrolysis. Green Chem.

[187] Zhang M, Resende FLP, Moutsoglou A, Raynie DE (2012). Pyrolysis of lignin extracted from prairie cordgrass, aspen, and Kraft lignin by Py-GC/MS and TGA/FTIR. J Anal Appl Pyrol.

[188] Fan Y, Zhang Z, Wang Z, Yu H, Kong X, Li P (2022). Radical footprinting and regularity revealing during the pyrolysis of technical lignins. Bioresour Technol.

[189] Demuner IF, Gomes FJB, Coura MR, Gomes JS, Demuner AJ, Carvalho AMML (2021). Determination of chemical modification of eucalypt kraft lignin after thermal treatment by Py-GC-MS. J Anal Appl Pyrol.

[190] Cao X, Shao L, Huang W, Wang C, Mao J, Xu F (2021). Thermal degradation of lignins fractionated by gradient acid precipitation. J Anal Appl Pyrol.

[191] Shimizu S, Posoknistakul P, Akiyama T, Yokoyama T, Matsumoto Y (2018). Effects of aromatic ring type on reactions subsequent to the β-O-4 bond cleavage of non-phenolic lignin model compounds under alkaline pulping conditions. J Wood Sci.

[192] Yu Q, Wang Y, Chen X, Wang F, Tian X, Gao Y (2021). Deep eutectic solvent assists *Bacillus australimaris* to transform alkali lignin waste into small aromatic compounds. J Clean Prod.

[193] Chandra R, Singh S, Krishna Reddy MM, Purohit HJ, Kapley A (2008). Isolation and characterization of bacterial strains *Paenibacillus* sp. and *Bacillus* sp. for kraft lignin decolorization from pulp paper mill waste. J Gen Appl Microbiol.

[194] Guo D-L, Wu S-B, Liu B, Yin X-L, Yang Q (2012). Catalytic effects of NaOH and Na_2_CO_3_ additives on alkali lignin pyrolysis and gasification. Appl Energy.

[195] Sun R, Tomkinson J, Bolton J (1999). Effects of precipitation pH on the physico-chemical properties of the lignins isolated from the black liquor of oil palm empty fruit bunch fibre pulping. Polym Degrad Stabil.

[196] Khanh Tran Q, Vu Ly H, Tae Hwang H, Kim J, Kim S-S (2022). Study on pyrolysis of Organosolv lignin impregnated with alkali and alkaline earth metals: kinetics, thermodynamics, and product characterization. Fuel.

[197] Quyn DM, Wu H, Bhattacharya SP, Li C-Z (2002). Volatilisation and catalytic effects of alkali and alkaline earth metallic species during the pyrolysis and gasification of Victorian brown coal. Part II. Effects of chemical form and valence. Fuel.

[198] Guo D-L, Yuan H-Y, Yin X-L, Wu C-Z, Wu S-B, Zhou Z-Q (2014). Effects of chemical form of sodium on the product characteristics of alkali lignin pyrolysis. Bioresour Technol.

[199] Dalluge DL, Kim KH, Brown RC (2017). The influence of alkali and alkaline earth metals on char and volatile aromatics from fast pyrolysis of lignin. J Anal Appl Pyrol.

[200] Zabeti M, Nguyen TS, Lefferts L, Heeres HJ, Seshan K (2012). In situ catalytic pyrolysis of lignocellulose using alkali-modified amorphous silica alumina. Bioresour Technol.

[201] Nguyen TS, Zabeti M, Lefferts L, Brem G, Seshan K (2013). Conversion of lignocellulosic biomass to green fuel oil over sodium based catalysts. Bioresour Technol.

[202] Lv D, Xu M, Liu X, Zhan Z, Li Z, Yao H (2010). Effect of cellulose, lignin, alkali and alkaline earth metallic species on biomass pyrolysis and gasification. Fuel Process Technol.

[203] Quyn DM, Wu H, Hayashi J-I, Li C-Z (2003). Volatilisation and catalytic effects of alkali and alkaline earth metallic species during the pyrolysis and gasification of Victorian brown coal. Part IV. Catalytic effects of NaCl and ion-exchangeable Na in coal on char reactivity. Fuel.

[204] Biswas B, Singh R, Kumar J, Khan AA, Krishna BB, Bhaskar T (2016). Slow pyrolysis of prot, alkali and dealkaline lignins for production of chemicals. Bioresour Technol.

[205] Guo D, Wu S, Lyu G, Guo H (2017). Effect of molecular weight on the pyrolysis characteristics of alkali lignin. Fuel.

[206] Pongchaiphol S, Suriyachai N, Hararak B, Raita M, Laosiripojana N, Champreda V (2022). Physicochemical characteristics of organosolv lignins from different lignocellulosic agricultural wastes. Int J Biol Macromol.

[207] Cheng X-C, Guo X-R, Qin Z, Wang X-D, Liu H-M, Liu Y-L (2020). Structural features and antioxidant activities of Chinese quince (*Chaenomeles sinensis*) fruits lignin during auto-catalyzed ethanol organosolv pretreatment. Int J Biol Macromol.

[208] Sheng Y, Ma Z, Wang X, Han Y (2022). Ethanol organosolv lignin from different agricultural residues: toward basic structural units and antioxidant activity. Food Chem.

[209] Parchami M, Agnihotri S, Taherzadeh MJ (2022). Aqueous ethanol organosolv process for the valorization of Brewer’s spent grain (BSG). Bioresour Technol.

[210] Warner G, Hansen TS, Riisager A, Beach ES, Barta K, Anastas PT (2014). Depolymerization of organosolv lignin using doped porous metal oxides in supercritical methanol. Bioresour Technol.

[211] Ramakoti B, Dhanagopal H, Deepa K, Rajesh M, Ramaswamy S, Tamilarasan K (2019). Solvent fractionation of organosolv lignin to improve lignin homogeneity: structural characterization. Bioresour Technol Rep.

[212] Laurichesse S, Avérous L (2014). Chemical modification of lignins: towards biobased polymers. Prog Polym Sci.

[213] Tolbert A, Akinosho H, Khunsupat R, Naskar AK, Ragauskas AJ (2014). Characterization and analysis of the molecular weight of lignin for biorefining studies. Biofuel Bioprod Biorefin.

[214] Hong S, Shen XJ, Pang B, Xue Z, Cao XF, Wen JL (2020). In-depth interpretation of the structural changes of lignin and formation of diketones during acidic deep eutectic solvent pretreatment. Green Chem.

[215] Hu B, Zhang B, Xie W-L, Jiang X-Y, Liu J, Lu Q (2020). Recent progress in quantum chemistry modeling on the pyrolysis mechanisms of lignocellulosic biomass. Energy Fuel.

[216] Soongprasit K, Sricharoenchaikul V, Atong D (2020). Phenol-derived products from fast pyrolysis of organosolv lignin. Energy Rep.

[217] Harman-Ware AE, Crocker M, Kaur AP, Meier MS, Kato D, Lynn B (2013). Pyrolysis-GC/MS of sinapyl and coniferyl alcohol. J Anal Appl Pyrol.

[218] Asmadi M, Kawamoto H, Saka S (2011). Thermal reactions of guaiacol and syringol as lignin model aromatic nuclei. J Anal Appl Pyrol.

[219] Dorrestijn E, Mulder P (1999). The radical-induced decomposition of 2-methoxyphenol. J Chem Soc Perkin Trans 2.

[220] Wang W, Liu Y, Wang Y, Liu L, Hu C (2022). The influence of solvent on the pyrolysis of organosolv lignins extracted from willow. Energy Convers Manag X.

[221] Chen D, Cen K, Zhuang X, Gan Z, Zhou J, Zhang Y (2022). Insight into biomass pyrolysis mechanism based on cellulose, hemicellulose, and lignin: evolution of volatiles and kinetics, elucidation of reaction pathways, and characterization of gas, biochar and bio-oil. Combust Flame.

[222] Wang C, Xia S, Cui C, Kang S, Zheng A, Yu Z (2022). Investigation into the correlation between the chemical structure of lignin and its temperature-dependent pyrolytic product evolution. Fuel.

[223] Lv X, Li Q, Jiang Z, Wang Y, Li J, Hu C (2018). Structure characterization and pyrolysis behavior of organosolv lignin isolated from corncob residue. J Anal Appl Pyrol.

[224] Chen Z, Bai X, Lusi A, Wan C (2018). High-solid lignocellulose processing enabled by natural deep eutectic solvent for lignin extraction and industrially relevant production of renewable chemicals. ACS Sustain Chem Eng.

[225] Li T, Lyu G, Liu Y, Lou R, Lucia LA, Yang G (2017). Deep eutectic solvents (DESs) for the isolation of willow lignin (*Salix matsudana* cv. Zhuliu). Int J Mol Sci.

[226] Liu X, Li T, Wu S, Ma H, Yin Y (2020). Structural characterization and comparison of enzymatic and deep eutectic solvents isolated lignin from various green processes: toward lignin valorization. Bioresour Technol.

[227] Xu H, Kong Y, Peng J, Song X, Liu Y, Su Z (2021). Comprehensive analysis of important parameters of choline chloride-based deep eutectic solvent pretreatment of lignocellulosic biomass. Bioresour Technol.

[228] Kim J-Y, Choi JW (2019). Effect of molecular size of lignin on the formation of aromatic hydrocarbon during zeolite catalyzed pyrolysis. Fuel.

[229] Li T, Yin Y, Wu S, Du X (2022). Effect of deep eutectic solvents-regulated lignin structure on subsequent pyrolysis products selectivity. Bioresour Technol.

[230] Jampa S, Puente-Urbina A, Ma Z, Wongkasemjit S, Luterbacher JS, Van Bokhoven JA (2019). Optimization of lignin extraction from pine wood for fast pyrolysis by using a γ-valerolactone-based binary solvent system. ACS Sustain Chem Eng.

[231] Wang S, Ru B, Lin H, Sun W, Luo Z (2015). Pyrolysis behaviors of four lignin polymers isolated from the same pine wood. Bioresour Technol.

[232] Li S, Luo Z, Wang W, Lu K, Yang Y, Liang X (2020). Characterization of pyrolytic lignin and insight into its formation mechanisms using novel techniques and DFT method. Fuel.

[233] Wang S, Ru B, Dai G, Shi Z, Zhou J, Luo Z (2017). Mechanism study on the pyrolysis of a synthetic β-O-4 dimer as lignin model compound. Proc Combust Inst.

[234] Li TF, Lyu G, Saeed HAM, Liu Y, Wu YL, Yang GH (2018). Analytical pyrolysis characteristics of enzymatic/mild acidolysis lignin (EMAL). BioResources.

[235] Dai L, Wang Y, Liu Y, Ruan R, He C, Duan D (2019). Bridging the relationship between hydrothermal pretreatment and co-pyrolysis: effect of hydrothermal pretreatment on aromatic production. Energy Convers Manag.

[236] Li X-Y, Guo T-S, Li M-F, Peng F (2021). Comparison of structure, thermal stability, and pyrolysis products of lignin extracted with ChCl-formic acid/lactic acid systems. J Mater Res Technol.

[237] Shen X-J, Chen T, Wang H-M, Mei Q, Yue F, Sun S (2020). Structural and morphological transformations of lignin macromolecules during bio-based deep eutectic solvent (DES) pretreatment. ACS Sustain Chem Eng.

[238] Wen JL, Xue BL, Sun SL, Sun RC (2013). Quantitative structural characterization and thermal properties of birch lignins after auto-catalyzed organosolv pretreatment and enzymatic hydrolysis. J Chem Technol Biotechnol.

[239] Ma Z, Sun Q, Ye J, Yao Q, Zhao C (2016). Study on the thermal degradation behaviors and kinetics of alkali lignin for production of phenolic-rich bio-oil using TGA-FTIR and Py-GC/MS. J Anal Appl Pyrol.

[240] Li H, Li X, You T, Li D, Nawaz H, Zhang X (2021). Insights into alkaline choline chloride-based deep eutectic solvents pretreatment for *Populus deltoides*: lignin structural features and modification mechanism. Int J Biol Macromol.

[241] Tan YT, Chua ASM, Ngoh GC (2020). Evaluation on the properties of deep eutectic solvent-extracted lignin for potential aromatic bio-products conversion. Ind Crop Prod.

[242] Wang T, Ye X, Yin J, Jin Z, Lu Q, Zheng Z (2014). Fast pyrolysis product distribution of biopretreated corn stalk by methanogen. Bioresour Technol.

[243] Yu H, Liu F, Ke M, Zhang X (2015). Thermogravimetric analysis and kinetic study of bamboo waste treated by *Echinodontium taxodii* using a modified three-parallel-reactions model. Bioresour Technol.

[244] Ke J, Singh D, Yang X, Chen S (2011). Thermal characterization of softwood lignin modification by termite *Coptotermes formosanus* (Shiraki). Biomass Bioenergy.

[245] Yan K, Liu F, Chen Q, Ke M, Huang X, Hu W (2016). Pyrolysis characteristics and kinetics of lignin derived from enzymatic hydrolysis residue of bamboo pretreated with white-rot fungus. Biotechnol Biofuel.

[246] Kong W, Fu X, Wang L, Alhujaily A, Zhang JL, Ma FY (2017). A novel and efficient fungal delignification strategy based on versatile peroxidase for lignocellulose bioconversion. Biotechnol Biofuel.

[247] Shi L, Yu H, Dong T, Kong W, Ke M, Ma F (2014). Biochemical and molecular characterization of a novel laccase from selective lignin-degrading white-rot fungus *Echinodontium taxodii* 2538. Process Biochem.

[248] Xu Z, Peng L, Zhai R, Wen Z, Jin M (2019). Recent advances in lignin valorization with bacterial cultures: microorganisms, metabolic pathways, and bio-products. Biotechnol Biofuel.

[249] Wang L, Ni H, Zhang J, Shi Q, Zhang R, Yu H (2020). Enzymatic treatment improves fast pyrolysis product selectivity of softwood and hardwood lignin. Sci Total Environ.

[250] Ma F, Huang X, Ke M, Shi Q, Chen Q, Shi C (2017). Role of selective fungal delignification in overcoming the saccharification recalcitrance of bamboo culms. ACS Sustain Chem Eng.

[251] Kozliak EI, Kubatova A, Artemyeva AA, Nagel E, Zhang C, Rajappagowda RB (2016). Thermal liquefaction of lignin to aromatics: efficiency, selectivity, and product analysis. ACS Sustain Chem Eng.

[252] Wang C, Ouyang XH, Su SS, Liang X, Zhang C, Wang WY (2016). Effect of sulfonated lignin on enzymatic activity of the ligninolytic enzymes C alpha-dehydrogenase LigD and beta-etherase LigF. Enzyme Microb Technol.

[253] Brebu M, Vasile C (2010). Thermal degradation of lignin—a review. Cell Chem Technol.

[254] Venkatesagowda B, Dekker RFH (2020). Enzymatic demethylation of Kraft lignin for lignin-based phenol-formaldehyde resin applications. Biomass Convers Biorefin.

[255] Majeke BM, Collard FX, Tyhoda L, Görgens JF (2022). The application of enzymatic pretreatment with subsequent pyrolysis to improve the production of phenols from selected industrial (technical) lignins. Waste Biomass Valoriz.

[256] Majeke BM, Collard FX, Tyhoda L, Görgens JF (2021). The synergistic application of quinone reductase and lignin peroxidase for the deconstruction of industrial (technical) lignins and analysis of the degraded lignin products. Bioresour Technol.

[257] Yan K, Liu F, Chen Q, Ke M, Huang X, Hu W (2016). Pyrolysis characteristics and kinetics of lignin derived from enzymatic hydrolysis residue of bamboo pretreated with white-rot fungus. Biotechnol Biofuel.

[258] Chiappe C, Pieraccini D (2005). Ionic liquids: solvent properties and organic reactivity. J Phys Org Chem.

[259] Zhang Q, Hu J, Lee D-J (2017). Pretreatment of biomass using ionic liquids: research updates. Renew Energy.

[260] Feng Y, Li Q, Kang G, Ji G, Tang Y, Tu J (2016). Aqueous two-phase/reverse micelle continuous process for recycling and simultaneous purification of polar ionic liquid from enzymatic hydrolysate. J Chem Technol Biotech.

[261] Lynam JG, Chow GI, Coronella CJ, Hiibel SR (2016). Ionic liquid and water separation by membrane distillation. Chem Eng J.

[262] Swatloski RP, Spear SK, Holbrey JD, Rogers RD (2002). Dissolution of cellose with ionic liquids. J Am Chem Soc.

[263] Carneiro AP, Rodriguez O, Macedo EA (2017). Dissolution and fractionation of nut shells in ionic liquids. Bioresour Technol.

[264] da Costa Lopes AM, João KG, Rubik DF, Bogel-Łukasik E, Duarte LC, Andreaus J (2013). Pre-treatment of lignocellulosic biomass using ionic liquids: wheat straw fractionation. Bioresour Technol.

[265] Khan AS, Man Z, Bustam MA, Kait CF, Ullah Z, Nasrullah A (2016). Kinetics and thermodynamic parameters of ionic liquid pretreated rubber wood biomass. J Mol Liq.

[266] Xue Z, Zhao X, Sun R-C, Mu T (2016). Biomass-derived γ-valerolactone-based solvent systems for highly efficient dissolution of various lignins: dissolution behavior and mechanism study. ACS Sustain Chem Eng.

[267] Pin TC, Nascimento VM, Costa AC, Pu Y, Ragauskas AJ, Rabelo SC (2020). Structural characterization of sugarcane lignins extracted from different protic ionic liquid pretreatments. Renew Energy.

[268] Usmani Z, Sharma M, Gupta P, Karpichev Y, Gathergood N, Bhat R (2020). Ionic liquid based pretreatment of lignocellulosic biomass for enhanced bioconversion. Bioresour Technol.

[269] Muhammad N, Man Z, Mutalib MA, Bustam MA, Wilfred CD, Khan AS (2015). Dissolution and separation of wood biopolymers using ionic liquids. ChemBioEng Rev.

[270] Yu W, Lei Z, Shui H, Ren S, Wang Z, Kang S (2017). Effect of ionic liquid 1-butyl-3-methyl-imidazolium dihydrogen phosphate pretreatment on pyrolysis of lignin. Coke Chem.

[271] Rashid T, Sher F, Khan AS, Khalid U, Rasheed T, Iqbal HMN (2021). Effect of protic ionic liquid treatment on the pyrolysis products of lignin extracted from oil palm biomass. Fuel.

[272] Sher F, Pans MA, Sun C, Snape C, Liu H (2018). Oxy-fuel combustion study of biomass fuels in a 20 kWth fluidized bed combustor. Fuel.

[273] Guida M, Bouaik H, Tabal A, Hannioui A, Solhy A, Barakat A (2016). Thermochemical treatment of olive mill solid waste and olive mill wastewater. J Therm Anal Calorim.

[274] Rashid T, Gnanasundaram N, Appusamy A, Kait CF, Thanabalan M (2018). Enhanced lignin extraction from different species of oil palm biomass: kinetics and optimization of extraction conditions. Ind Crop Prod.

[275] Mohtar S, Busu TTM, Noor AM, Shaari N, Yusoff N, Bustam M (2015). Extraction and characterization of lignin from oil palm biomass via ionic liquid dissolution and non-toxic aluminium potassium sulfate dodecahydrate precipitation processes. Bioresour Technol.

[276] Hu Y, Wang Z, Cheng X, Ma C (2018). Non-isothermal TGA study on the combustion reaction kinetics and mechanism of low-rank coal char. RSC Adv.

[277] Mabuda A, Mamphweli N, Meyer E (2016). Model free kinetic analysis of biomass/sorbent blends for gasification purposes. Renew Sustain Energy Rev.

[278] Muhammad N, Omar WN, Man Z, Bustam MA, Rafiq S, Uemura Y (2012). Effect of ionic liquid treatment on pyrolysis products from bamboo. Ind Eng Chem Res.

[279] Lei Z, Dong L, Hu Z, Li Z, Yan J, Shui H (2019). Pyrolysis of acidic ionic liquid 1-sulfonic acid butyl-3-methylimidazolium trifluoromethanesulfonate-treated lignin for phenolics production. Energy Fuel.

[280] Lei Z-P, Hu Z-Q, Shui H-F, Ren S-B, Wang Z-C, Kang S-G (2015). Pyrolysis of lignin following ionic liquid pretreatment at low temperature. Fuel Process Technol.

[281] Sun H, Luo Z, Wang W, Li S, Xue S (2021). Porosity roles of micro-mesostructured ZSM-5 in catalytic fast pyrolysis of cellulolytic enzyme lignin for aromatics. Energy Convers Manag.

[282] Soongprasit K, Sricharoenchaikul V, Atong D (2021). Selective aromatic production from fast pyrolysis of sugarcane bagasse lignin over ZSM-5 catalyst. Energy Rep.

[283] Zhang H, Xiao R, Wang D, Zhong Z, Song M, Pan Q (2009). Catalytic fast pyrolysis of biomass in a fluidized bed with fresh and spent fluidized catalytic cracking (FCC) catalysts. Energy Fuel.

[284] Wang S, Li Z, Yi W, Fu P, Zhang A, Bai X (2021). Renewable aromatic hydrocarbons production from catalytic pyrolysis of lignin with Al-SBA-15 and HZSM-5: synergistic effect and coke behaviour. Renew Energy.

[285] Kumar A, Kumar A, Kumar J, Bhaskar T (2019). Catalytic pyrolysis of soda lignin over zeolites using pyrolysis gas chromatography–mass spectrometry. Bioresour Technol.

[286] Tang S, Zhang C, Xue X, Pan Z, Wang D, Zhang R (2019). Catalytic pyrolysis of lignin over hierarchical HZSM-5 zeolites prepared by post-treatment with alkaline solutions. J Anal Appl Pyrol.

[287] Balasundram V, Ibrahim N, Kasmani RM, Hamid MKA, Isha R, Hasbullah H (2017). Thermogravimetric catalytic pyrolysis and kinetic studies of coconut copra and rice husk for possible maximum production of pyrolysis oil. J Clean Prod.

[288] Jin T, Wang H, Peng J, Wu Y, Huang Z, Tian X (2022). Catalytic pyrolysis of lignin with metal-modified HZSM-5 as catalysts for monocyclic aromatic hydrocarbons production. Fuel Process Technol.

[289] Yang M, Shao J, Yang Z, Yang H, Wang X, Wu Z (2019). Conversion of lignin into light olefins and aromatics over Fe/ZSM-5 catalytic fast pyrolysis: significance of Fe contents and temperature. J Anal Appl Pyrol.

[290] Lago A, Hernando H, Moreno JM, Serrano DP, Fermoso J (2021). Valorisation of a lignin-rich residue via catalytic pyrolysis over ZrO_2_/ZSM-5 technical catalyst. Fuel Process Technol.

[291] Zhang H, Luo B, Wu K, Wu H, Yu J, Wang S (2022). Enhancing aromatic yield from catalytic pyrolysis of Ca^2+^-loaded lignin coupled with metal-modified HZSM-5. Appl Energy Combust Sci.

[292] Xue S, Luo Z, Wang W, Li S, Sun H, Zhou Q (2020). Preparation of aromatics from catalytic pyrolysis of enzymatic lignin over double-layer metal supported core–shell catalyst. J Anal Appl Pyrol.

[293] Wang S, Li Z, Yi W, Fu P, Bai X (2022). Regulating aromatic hydrocarbon components from catalytic pyrolysis of corn cob lignin with a tailored HZSM-5@Al-SBA-15 hierarchical zeolite. Ind Crop Prod.

[294] Li W, Wang D, Zhu Y, Chen J, Lu Y, Li S (2020). Efficient ex-situ catalytic upgrading of biomass pyrolysis vapors to produce methylfurans and phenol over bio-based activated carbon. Biomass Bioenergy.

[295] Yang H, Chen Z, Chen W, Chen Y, Wang X, Chen H (2020). Role of porous structure and active O-containing groups of activated biochar catalyst during biomass catalytic pyrolysis. Energy.

[296] Yang H, Han T, Shi Z, Sun Y, Jiang J, Sandström L (2022). In situ catalytic fast pyrolysis of lignin over biochar and activated carbon derived from the identical process. Fuel Process Technol.

[297] Zhang H, Luo B, Wu K, Zhao B, Yu J, Wang S (2022). Ex-situ catalytic pyrolysis of lignin using lignin-carbon catalyst combined with HZSM-5 to improve the yield of high-quality liquid fuels. Fuel.

[298] Yu J, Luo B, Wang Y, Wang S, Wu K, Liu C (2022). An efficient way to synthesize biomass-based molybdenum carbide catalyst via pyrolysis carbonization and its application for lignin catalytic pyrolysis. Bioresour Technol.

[299] Torri C, Reinikainen M, Lindfors C, Fabbri D, Oasmaa A, Kuoppala E (2010). Investigation on catalytic pyrolysis of pine sawdust: catalyst screening by Py-GC-MIP-AED. J Anal Appl Pyrol.

[300] Zhang X, Sun L, Chen L, Xie X, Zhao B, Si H (2014). Comparison of catalytic upgrading of biomass fast pyrolysis vapors over CaO and Fe(III)/CaO catalysts. J Anal Appl Pyrol.

[301] Guan W, Chen X, Jin S, Li C, Tsang C-W, Liang C (2017). Highly stable Nb_2_O_5_–Al_2_O_3_ composites supported Pt catalysts for hydrodeoxygenation of diphenyl ether. Ind Eng Chem Res.

[302] Kong L, Zhang L, Gu J, Gou L, Xie L, Wang Y (2020). Catalytic hydrotreatment of kraft lignin into aromatic alcohols over nickel-rhenium supported on niobium oxide catalyst. Bioresour Technol.

[303] Li S, Luo Z, Wang W, Sun H, Xie J, Liang X (2020). Catalytic fast pyrolysis of enzymatic hydrolysis lignin over Lewis-acid catalyst niobium pentoxide and mechanism study. Bioresour Technol.

[304] Xin Y, Dong L, Guo Y, Liu X, Hu Y, Wang Y (2019). Correlation of the catalytic performance with Nb_2_O_5_ surface properties in the hydrodeoxygenation of lignin model compound. J Catal.

[305] Ding Y, Wang S, Zhang L, Chen Z, Wang M, Wang S (2017). A facile method to promote LaMnO_3_ perovskite catalyst for combustion of methane. Catal Commun.

[306] Han H, Ge Q, Zhang M, Chen Y, Wang H, Zhang Y (2022). Production of phenolic compounds in catalytic pyrolysis of bagasse lignin in the presence of Ca_0.5_Pr_0.5_FeO_3_. J Rare Earth.

[307] Wang H, Han H, Sun E, Zhang Y, Li J, Chen Y (2019). Production of aryl oxygen-containing compounds via catalytic pyrolysis of bagasse lignin over La_0.8_M_0.2_FeO_3_ (M=La, Ca, Sr, Ba). J Anal Appl Pyrol.

[308] Wang H, Han H, Sun E, Zhang Y, Li J, Chen Y (2019). Production of aryl oxygen-containing compounds from catalytic pyrolysis of bagasse lignin over LaTi_x_Fe_1−x_O_3_. Chin J Chem Eng.

[309] Wang H, Han H, Sun E, Zhang Y, Li J, Chen Y (2020). Production of aryl oxygen-containing compounds by catalytic pyrolysis of bagasse lignin over LaTi_0.2_Fe_0.8_O_3_ prepared by different methods. J Rare Earth.

[310] Wang H, Han H, Zhang Y, Li J, Chen Y, Song H (2019). Influence of calcination temperature for LaTi_0.2_Fe_0.8_O_3_ on catalytic pyrolysis of bagasse lignin. J Rare Earth.

[311] Yang H, Han T, Yang W, Sandström L, Jönsson PG (2022). Influence of the porosity and acidic properties of aluminosilicate catalysts on coke formation during the catalytic pyrolysis of lignin. J Anal Appl Pyrol.

[312] Margellou AG, Lazaridis PA, Charisteidis ID, Nitsos CK, Pappa CP, Fotopoulos AP (2021). Catalytic fast pyrolysis of beech wood lignin isolated by different biomass (pre)treatment processes: organosolv, hydrothermal and enzymatic hydrolysis. Appl Catal A-Gen.

[313] Jin T, Zhang D, Peng J, Wu Y, Ma J, Zhang J (2022). Pretreatment of HZSM-5 with organic alkali and cobalt: application in catalytic pyrolysis of lignin to produce monocyclic aromatic hydrocarbons. Fuel Process Technol.

[314] Zhao B, Wu K, Zhong L-P, Wei G, Hu Z-H, Zheng W-G (2021). Experimental study on catalytic pyrolysis of lignin under char and ZSM-5 for preparation of aromatics. J Fuel Chem Technol.

[315] Fan L, Zhang Y, Liu S, Zhou N, Chen P, Cheng Y (2017). Bio-oil from fast pyrolysis of lignin: effects of process and upgrading parameters. Bioresour Technol.

[316] Engamba Esso SB, Xiong Z, Chaiwat W, Kamara MF, Longfei X, Xu J (2022). Review on synergistic effects during co-pyrolysis of biomass and plastic waste: significance of operating conditions and interaction mechanism. Biomass Bioenergy.

[317] Patil V, Adhikari S, Cross P (2018). Co-pyrolysis of lignin and plastics using red clay as catalyst in a micro-pyrolyzer. Bioresour Technol.

[318] Kunwar B, Cheng HN, Chandrashekaran SR, Sharma BK (2016). Plastics to fuel: a review. Renew Sustain Energy Rev.

[319] Xiong S, Zhuo J, Zhou H, Pang R, Yao Q (2015). Study on the co-pyrolysis of high density polyethylene and potato blends using thermogravimetric analyzer and tubular furnace. J Anal Appl Pyrol.

[320] Sophonrat N, Sandström L, Johansson A-C, Yang W (2017). Co-pyrolysis of mixed plastics and cellulose: an interaction study by Py-GC×GC/MS. Energy Fuel.

[321] Yuan C, Wang S, Cao B, Hu Y, Abomohra AE-F, Wang Q (2019). Optimization of hydrothermal co-liquefaction of seaweeds with lignocellulosic biomass: merging 2nd and 3rd generation feedstocks for enhanced bio-oil production. Energy.

[322] Önal E, Uzun BB, Pütün AE (2014). Bio-oil production via co-pyrolysis of almond shell as biomass and high density polyethylene. Energy Convers Manag.

[323] Zhong S, Zhang B, Liu C, Shujaa AA (2021). Mechanism of synergistic effects and kinetics analysis in catalytic co-pyrolysis of water hyacinth and HDPE. Energy Convers Manag.

[324] Stančin H, Šafář M, Růžičková J, Mikulčić H, Raclavská H, Wang X (2021). Co-pyrolysis and synergistic effect analysis of biomass sawdust and polystyrene mixtures for production of high-quality bio-oils. Process Saf Environ.

[325] Tao L, Ma X, Ye L, Jia J, Wang L, Ma P (2021). Interactions of lignin and LDPE during catalytic co-pyrolysis: thermal behavior and kinetics study by TG-FTIR. J Anal Appl Pyrol.

[326] Xu L, He Z, Zhang H, Wu S, Dong C, Fang Z (2021). Production of aromatic amines via catalytic co-pyrolysis of lignin and phenol-formaldehyde resins with ammonia over commercial HZSM-5 zeolites. Bioresour Technol.

[327] Ma C, Xie S, Kumagai S, Takahashi Y, Saito Y, Kameda T (2022). Synergistic effects during co-pyrolysis of milled wood lignin and polyolefins at the gas phase and liquid/solid phase contacting modes. Chem Eng J.

[328] Ke L, Wang Y, Wu Q, Zhou N, Dai L, Tian X (2022). Pressurized ex-situ catalytic co-pyrolysis of polyethylene and lignin: efficient BTEX production and process mechanism analysis. Chem Eng J.

[329] Wan Z, Wang S, Li Z, Yi W, Zhang A, Li Y (2022). Co-pyrolysis of lignin and spent bleaching clay: insight into the catalytic characteristic and hydrogen supply of spent bleaching clay. J Anal Appl Pyrol.

[330] Anuar Sharuddin SD, Abnisa F, Wan Daud WMA, Aroua MK (2016). A review on pyrolysis of plastic wastes. Energy Convers Manag.

[331] Zhao Z, Cannon FS, Nieto-Delgado C (2019). Synergistic interaction between lignin and collagen during co-pyrolysis. Carbon.

[332] Bai J, Chen X, Shao J, Jia C, Wang Q (2019). Study of breakage of main covalent bonds during co-pyrolysis of oil shale and alkaline lignin by TG-FTIR integrated analysis. J Energy Inst.

[333] Li C, Sun Y, Yi Z, Zhang L, Zhang S, Hu X (2022). Co-pyrolysis of coke bottle wastes with cellulose, lignin and sawdust: impacts of the mixed feedstock on char properties. Renew Energy.

[334] Fan Y, Yang B, Zhang B, Wu Z, Sun Z, Shang J (2021). Synergistic effects from fast co-pyrolysis of lignin with low-rank coal: on-line analysis of products distribution and fractal analysis on co-pyrolysis char. J Energy Inst.

[335] Bi H, Ni Z, Tian J, Jiang C, Sun H, Lin Q (2022). Influence of lignin on coal gangue pyrolysis and gas emission based on multi-lump parallel reaction model and principal component analysis. Sci Total Environ.

[336] Tumbalam Gooty A, Li D, Berruti F, Briens C (2014). Kraft-lignin pyrolysis and fractional condensation of its bio-oil vapors. J Anal Appl Pyrol.

[337] Xu R, Ferrante L, Briens C, Berruti F (2009). Flash pyrolysis of grape residues into biofuel in a bubbling fluid bed. J Anal Appl Pyrol.

[338] Deng J-J, Luo Z-J, Wang C, Zhu X-F (2021). Extraction of phenols from bio-oil aqueous fraction by hydrophobic ionic liquids. J Fuel Chem Technol.

[339] Yao T, Li H, Ren Y, Feng M, Hu Y, Yan H (2022). Extraction and recovery of phenolic compounds from aqueous solution by thermo-separating magnetic ionic liquid aqueous two-phase system. Sep Purif Technol.

[340] Xu D, Wang S, Zhang T, Peng L, Bing X, Zhang L (2022). Extraction and interaction insights for enhanced separation of phenolic compounds from model coal tar using a hydroxyl-functionalized ionic liquid. Chem Eng Res Des.

[341] Yang W, Wang X, Ni S, Liu X, Liu R, Hu C (2021). Effective extraction of aromatic monomers from lignin oil using a binary petroleum ether/dichloromethane solvent. Sep Purif Technol.

[342] Kariyawasam T, Doran GS, Howitt JA, Prenzler PD (2022). Polycyclic aromatic hydrocarbon contamination in soils and sediments: sustainable approaches for extraction and remediation. Chemosphere.

